# Discovery and characterization of functional modules associated with body weight in broilers

**DOI:** 10.1038/s41598-019-45520-5

**Published:** 2019-06-24

**Authors:** Eirini Tarsani, Andreas Kranis, Gerasimos Maniatis, Santiago Avendano, Ariadne L. Hager-Theodorides, Antonios Kominakis

**Affiliations:** 1 Department of Animal Science and Aquaculture, Agricultural University of Athens, Iera Odos 75, 11855 Athens, Greece; 2 Aviagen Ltd., Newbridge, Midlothian EH28 8SZ UK; 3 The Roslin Institute, University of Edinburgh, EH25 9RG Midlothian, United Kingdom

**Keywords:** Genomics, Animal breeding

## Abstract

Aim of the present study was to investigate whether body weight (BW) in broilers is associated with functional modular genes. To this end, first a GWAS for BW was conducted using 6,598 broilers and the high density SNP array. The next step was to search for positional candidate genes and QTLs within strong LD genomic regions around the significant SNPs. Using all positional candidate genes, a network was then constructed and community structure analysis was performed. Finally, functional enrichment analysis was applied to infer the functional relevance of modular genes. A total number of 645 positional candidate genes were identified in strong LD genomic regions around 11 genome-wide significant markers. 428 of the positional candidate genes were located within growth related QTLs. Community structure analysis detected 5 modules while functional enrichment analysis showed that 52 modular genes participated in developmental processes such as skeletal system development. An additional number of 14 modular genes (*GABRG1, NGF, APOBEC2, STAT5B, STAT3, SMAD4, MED1, CACNB1, SLAIN2, LEMD2, ZC3H18, TMEM132D, FRYL* and *SGCB*) were also identified as related to body weight. Taken together, current results suggested a total number of 66 genes as most plausible functional candidates for the trait examined.

## Introduction

Body weight (BW) is an economically important trait for the broiler industry. This trait also presents considerable biological interest as it is a typical complex (polygenic) trait. To date, the ChickenQTLdb^1^ contains over 7,812 QTL/SNP associations of which 3,582 are related to growth. Several genome wide association studies (GWAS) have already been performed for growth traits (e.g.^2,3^) in the species. The development of the chicken 600k SNP array^4^ facilitates efficient screening for causal loci and genes with relevance to target traits due to the uniform coverage across chromosomes and the inclusion of markers within coding regions. Despite the large number of findings by GWAS, understanding of the genetic architecture of BW in chicken remains limited^5^, since only a small number of positional candidate genes are confirmed as truly functionally relevant to the trait (e.g. *HDAC2* and *GNPDA2*^6,7^). The use of various Bioinformatics tools such as gene enrichment analysis^8^, pathway analysis^9^ and gene network analysis^10^ can tackle this problem and aid in identifying the most promising functional candidate genes for the trait under study. Moreover, applications such as GeneMANIA^11^ that is based on the guilt-by-association (GBA) principle^12^ may also facilitate the identification of true causative genetic variants. The GBA principle states that gene products, which are protein interaction partners, tend to be functionally related^13^. Furthermore, genes in protein–protein interaction networks (PPINs) are organized into densely linked clusters i.e. communities or modules^14^. Modules present a structurally independent gene sub-network with more interior connections and consist of proteins which have the same or similar biological function(s)^15^. Modules could be further distinguished in protein complexes and in dynamic functional modules. Protein complexes are formed by several proteins which interact at the same place and time while dynamic functional modules are composed of few proteins participating in a specific cellular function not necessarily at the same place and time^16^. Moreover, functional modules consist of one or multiple protein complexes participating in a common biological process^17^. Since modules do not emerge by chance, they can reveal interactions with biological importance within large PPINs^16,18^. The module-based approach has already been used to cluster genes into functional groups and to predict protein functions^19^. Investigation of functional modules has mainly been focused on human diseases such as obesity^20^, breast cancer^21,22^, coronary artery disease^23^ and asthma^24^. Apart from human, functional modules have been identified in other species as well, such as in *Mus musculus* for discrete and rhythmic forelimb movements in motor cortex^25^ and in *Gallus gallus* for muscle development and intramuscular fat accumulation at different post-hatching ages^26^.

Driven from findings in other species and traits, aim of the present study was first to investigate whether body weight in broilers is associated with functional modules and second to propose novel candidate genes for the trait in question.

## Results

### Significant SNPs and positional candidate genes

**Figure 1:**
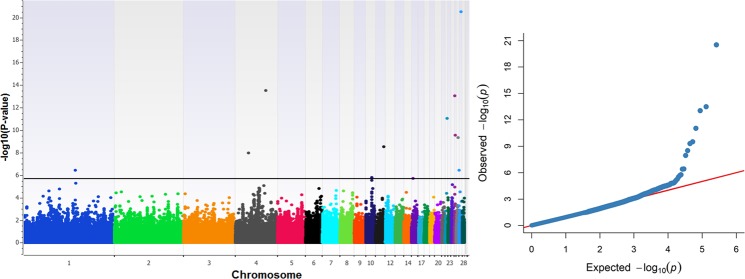
Manhattan plot (left) and quantile-quantile plot (right) for BW. Manhattan plot shows the −log_10_ (observed p-values) of the genome-wide SNPs (y-axis) across the 28 autosomes (x-axis), and the horizontal line denotes the genome-wide significant threshold. With regard to the Q-Q plot, the y-axis represents the observed −log_10_ (p-values) and the x-axis shows the expected −log_10_ (p-values). Manhattan plot was constructed with SNP & Variation Suite (version 8.8.1) software (Golden Helix: http://www.goldenhelix.com) while Q-Q plot with the CMplot package (https://github.com/YinLiLin/R-CMplot) in R (http://www.r-project.org/).

Figure 1 shows the Q-Q plot of the expected and the observed p values (−log_10_ p values) of all SNPs. The genomic inflation factor (λ) was also estimated as high as 0.93. According to Kang *et al*.^27^, λ values that lie outside of the conservative 95% confidence interval (0.992 to 1.008) denote dependency of SNPs. However, as the Q-Q plot clearly shows, there is no evidence of any systematic bias due to population structure or analytical approach in our case. As Yang *et al*.^28^ emphasize in their paper, it is reasonable to expect deviation(s) of λ from 1 for purely polygenic traits such as that examined here in the absence of any systematic bias. The Q-Q plot also shows that some SNPs depart from the expected probability and thus might be associated with the trait. These SNPs are also displayed in Fig. 1 in a form of a Manhattan plot.

Specifically, there were 12 SNPs detected, across nine autosomes (1, 4, 10, 11, 15, 22, 25, 26 and 27) reaching genome-wide significance (FDR p-value < 0.05). A detailed description of the significant (lead) SNPs is provided in Table 1. Table 2 displays the extent of genomic regions displaying strong LD (D′ > 0.8) around the lead markers that were searched for positional candidate genes. Note that marker *rs312758346* (GGA25) was omitted here as LD levels around this marker were below the threshold LD value (D′ < 0.8). In total, 645 positional candidate genes were identified within the searched genomic regions (Supplementary Table S1). From the candidate genes, n = 15 were microRNAs with 13 of them (*MIR6672, MIR1720, MIR7-2, MIR3529, MIR1571, MIR1560, MIR1785, MIR6662, MIR7454, MIR10A, MIR6663, MIR1735 and MIR6547*) published in the miRBase database (http://www.mirbase.org/) for the species. Moreover, 190 candidate genes were unannotated (LOC) resulting in a total number of 455 annotated positional candidate genes. The maximum number of candidate genes (n = 192) was identified in a region spanning 998.5 kb (average D′ = 0.98) around marker *rs315329074 on* GGA27. At the other extreme, the smallest number of candidate genes was identified for *rs316794400* within a narrow region spanning 26.6 kb (average D′ = 0.96) on GGA22. Six out of the 11 lead markers were located within annotated genes i.e. *SLAIN2* (GGA4)*, ZC3H18* (GGA11)*, TMEM132D* (GGA15)*, F-KER* (GGA25)*, LEMD2* (GGA26) and *CACNB1* (GGA27).

**Table 1:** Genome-wide significant SNPs (FDR p-value < 0.05) for BW.

SNP ID	GGA	Position (bp)^1^	−log_10_(p-value)	FDR p-value
*rs13923872*	1	112,741,685	6.415	0.0112
*rs312691174*	4	29,074,989	7.948	0.00037
*rs15608447*	4	66,885,210	13.489	4.25E-09
*rs318199727*	10	13,536,548	5.763	0.04111
*rs318098582*	11	18,651,449	8.513	0.00012
*rs317945754*	15	3,557,083	5.677	0.04594
*rs316794400*	22	4,594,855	11.033	6.07E-07
*rs317288536*	25	976,833	13.035	8.05E-09
*rs312758346*	25	2,412,866	9.517	1.59E-05
*rs317627533*	26	4,597,439	9.313	2.12E-05
*rs314452928*	27	104,022	6.398	0.0105
*rs315329074*	27	4,528,275	20.513	8.05E-16

^1^Positions are based on *Gallus gallus-5.0* genome assembly.

**Table 2:** Number of positional candidate genes and QTL/associations within the searched genomic regions (±maximum distance of the farest SNP being in strong LD (D′ >0.8) with the lead SNP; D′: average D′ values within the searched genomic region).

SNP ID	GGA	Position (bp)^1^	Searched genomic range around ‘lead’ SNP (±bp)	D′	Number of positional candidate genes	Number of QTL/associations
*rs13923872*	1	112,741,685	613,054	0.91	33	20
*rs312691174*	4	29,074,989	650,472	1	16	14
*rs15608447*	4	66,885,210	718,407	0.88	36	36
*rs318199727*	10	13,536,548	737,906	0.83	33	11
*rs318098582*	11	18,651,449	300,257	0.81	27	9
*rs317945754*	15	3,557,083	935,183	0.99	20	21
*rs316794400*	22	4,594,855	26,589	0.96	7	1
*rs317288536*	25	976,833	1,004,513	0.83	176	—
*rs317627533*	26	4,597,439	773,988	0.9	93	6
*rs314452928*	27	104,022	140,067	0.94	12	3
*rs315329074*	27	4,528,275	998,553	0.98	192	65

^1^Positions are based on *Gallus gallus-5.0* genome assembly.

### Reported QTL/associations

Table 2 shows the number of published QTL/associations reported within the searched genomic regions. A total of 186 QTL/associations related to growth traits or carcass traits (carcass weight, abdominal fat percentage, breast muscle percentage and average daily gain) were identified within the searched regions. QTL/associations were distributed across eight chromosomes (1, 4, 10, 11, 15, 22, 26 and 27) and a detailed description of the reported QTL can be found in Supplementary Table S2. Note that the searched region around *rs317288536 (*GGA25) is not reported to harbor any QTL/association (Table 2). Furthermore, the only QTL reported on GGA22 as well as two additional QTL on GGA26 and GGA27 could not be remapped in *Gallus gallus*−5.0 by the Genome remapping service tool from NCBI database. Nevertheless, based on the *Gallus gallus-4* genome assembly, the searched regions around *rs316794400, rs317627533* and *rs314452928* overlapped with three QTL (IDs: 95429, 30883 and 55944). The maximum number (n = 65) of QTL/associations was reported around *rs315329074* (GGA27) and the minimum number (n = 1) around *rs316794400 (*GGA22*)*. Nine out of the 12 lead SNPs on autosomes 1, 4, 10, 11, 15, 26 and 27 lie within 96 out of the 186 growth-related QTL (Supplementary Table S2). In addition, nearly all reported QTL on the searched regions on GGA4 (n = 49/50) and GGA11 (n = 9/9) contain at least one of the lead markers (*rs312691174*, *rs15608447* and *rs318098582* respectively).

**Figure 2:**
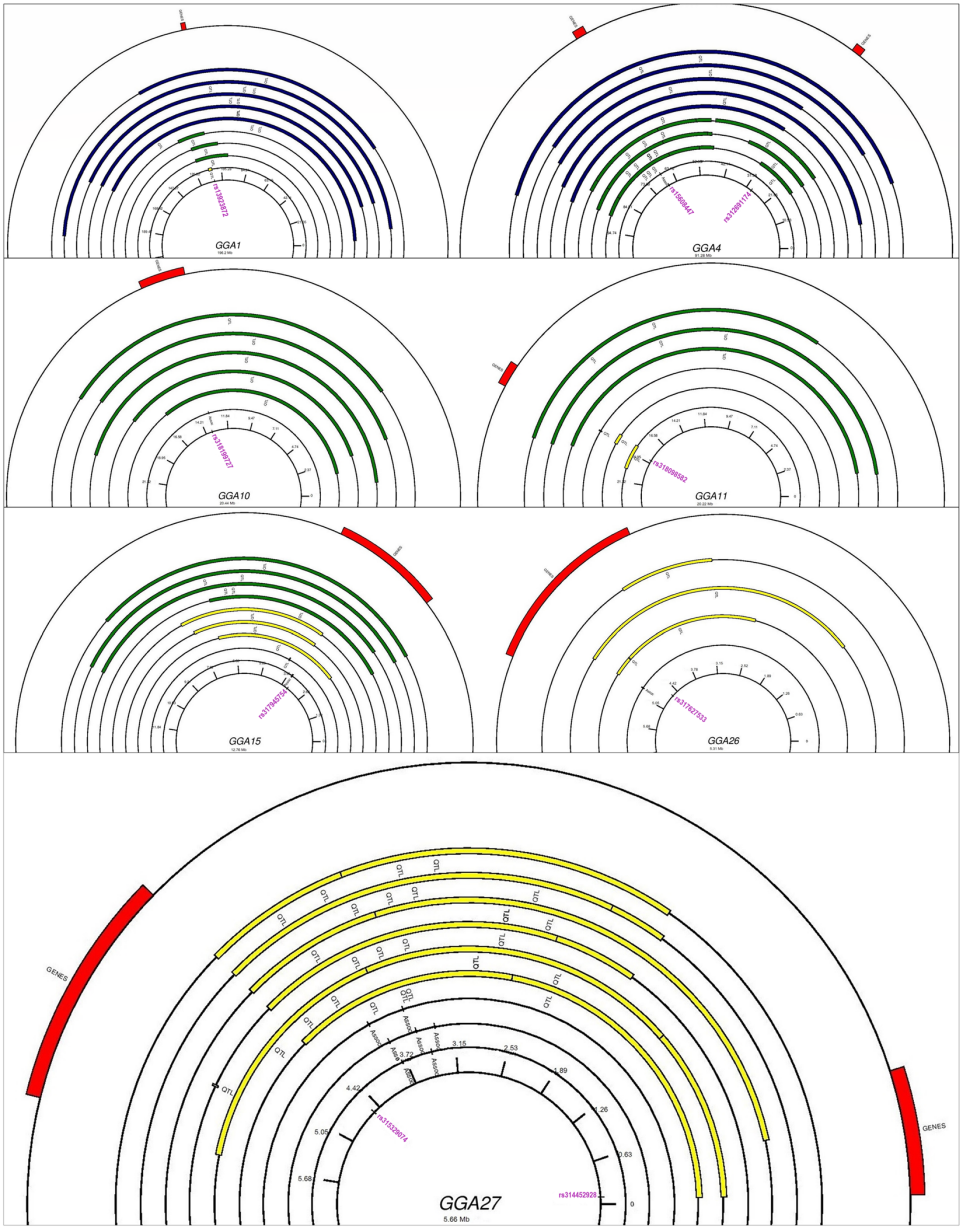
Circular chromosome maps for seven autosomes presenting combined data of reported QTL (n = 183) and positional candidate genes (n = 462). Blue color represents the extent of large sized QTL (50–196.2 Mb), green color the medium sized QTL (5–50 Mb) and the yellow color is indicative of the small QTL (0–5 Mb). Red color indicates the starting and ending positions of positional candidate genes. The position(s) of the significant SNPs (labeled in purple color) is also given. The figure was constructed using GenomeVx^87^.

We further sought to examine the locations of the positional candidate genes in the relation to the positions of the reported QTL. These results are illustrated in Fig. 2 in forms of circular maps for seven autosomes (1, 4, 10, 11, 15, 26 and 27). On GGA1, all 33 candidate genes (around *rs13923872*) are lying in a genomic region spanning from 2421 to 196203 kb where 17 relevant QTL have been reported. On GGA4, all 16 candidate genes around *rs312691174* are located within a region spanning from 4965 to 91268 kb, where all 14 published QTL reside. On the same autosome, all genes (n = 36) around the second significant marker (*rs15608447*) are located in a region spanning from 4965 to 91268 kb where 35 QTL relevant to body weight, liver weight, carcass weight, total white fat weight have been reported. On GGA10, the region spanning from 693 to 20423 kb around the ‘lead’ marker (*rs318199727)* harbours all the 33 candidate genes and overlaps with 6 growth related QTL. On GGA11, in a region spanning from 953 to 20209 kb around *rs318098582* there have been 7 reported QTL and 27 candidate genes identified. On GGA15, in a region spanning from 1932 to 10689 kb around *rs317945754)*, 6 QTL related to growth traits (visceral fat weight, abdominal fat weight and breast muscle weight) are reported and 20 candidate genes were identified. Moreover, on GGA26 *(rs317627533)*, 64 out of the 93 candidate genes lie in a narrow region (1264 to 4918 kb) where QTL associated with growth traits such as body weight and shank weight, are reported. On GGA27 in a regions spanning from 55 to 4520 kb around *rs314452928*, 2 related QTL were identified including 7 out of the 12 candidate genes. All 192 genes around the second marker (*rs315329074*) on GGA27 were located within one published QTL spanning 3788 to 5630 kb that has been associated with thigh percentage. In total, 428 out of the 462 positional candidate genes (genes on GGA22 and GGA25 were not included here) were located within regions with reported QTL/associations.

### Detection of community structure

**Figure 3:**
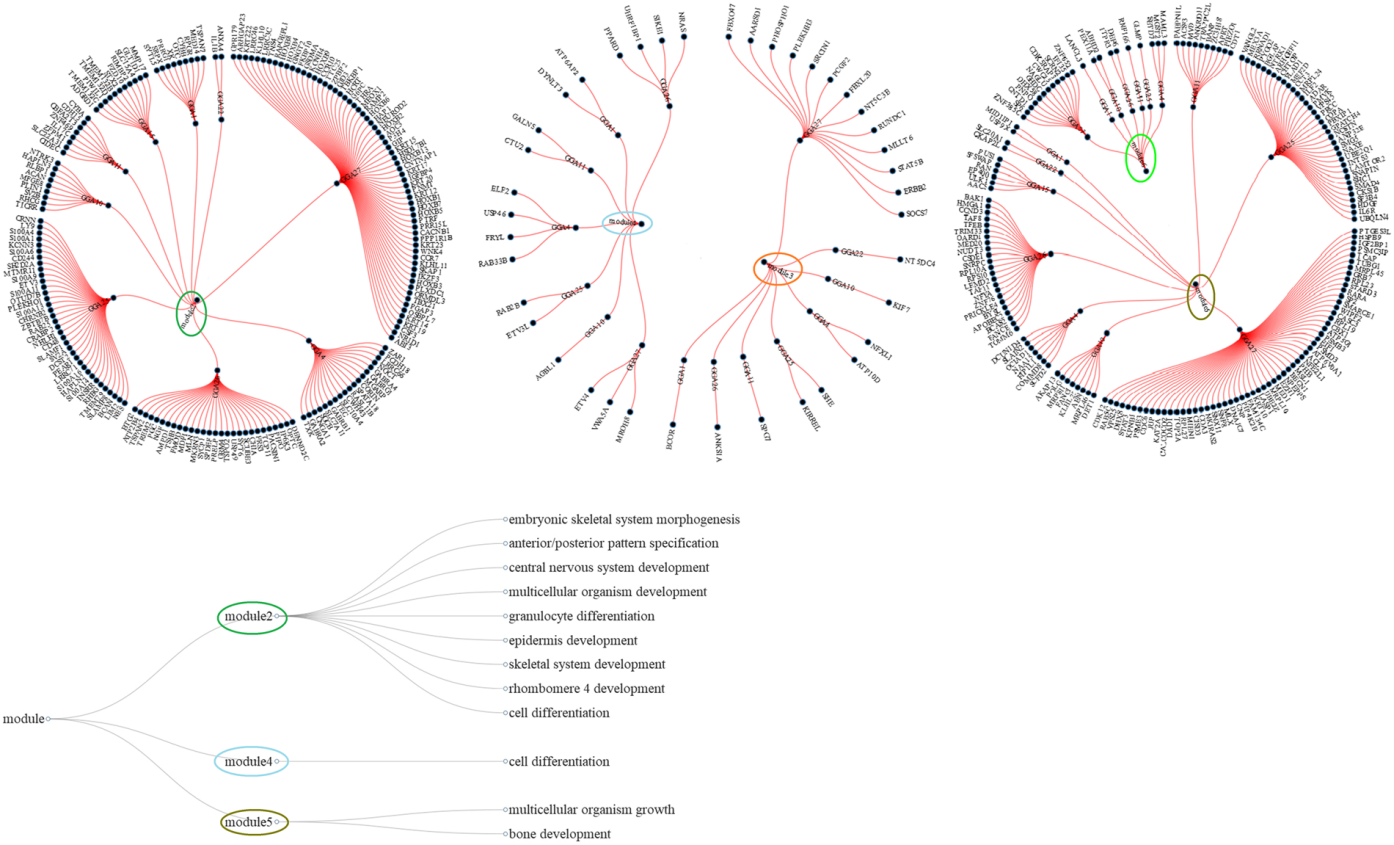
Network modules along with the significantly enriched developmental processes per module. The five modules are presented in the three radial networks (on the top) as circles/ellipses with different color together with their member genes and the corresponding chromosomes. The diagonal network at the bottom provides the significantly enriched developmental processes per module. Figure was constructed using the data.tree and networkD3 packages in R (http://www.r-project.org/)^.^

A network including 402 genes (nodes) and 5294 interactions (edges) was generated. Note that for *APOA1BP* and *LOH11CR2A* genes the homologous human gene descriptions (*NAXE* and *VWA5A)* were used, respectively. Community structure analysis detected 5 modules, formed by 401 genes (see Supplementary Table S3). One more module was also detected but this was consisted by only one gene (*NIPAL1*). Thus it cannot be considered as a typical module. Note that this gene network had a strong community structure as indicated by the high (0.59) estimated modularity value^29^. Distribution of the 401 genes across the 5 modules is displayed in Fig. 3. Module_2 consisted of 187 genes, module_3 of 22 genes, module_4 of 18 genes, module_5 of 152 genes and module_6 of 22 genes.

### Functional enrichment analysis per module

**Figure 4:**
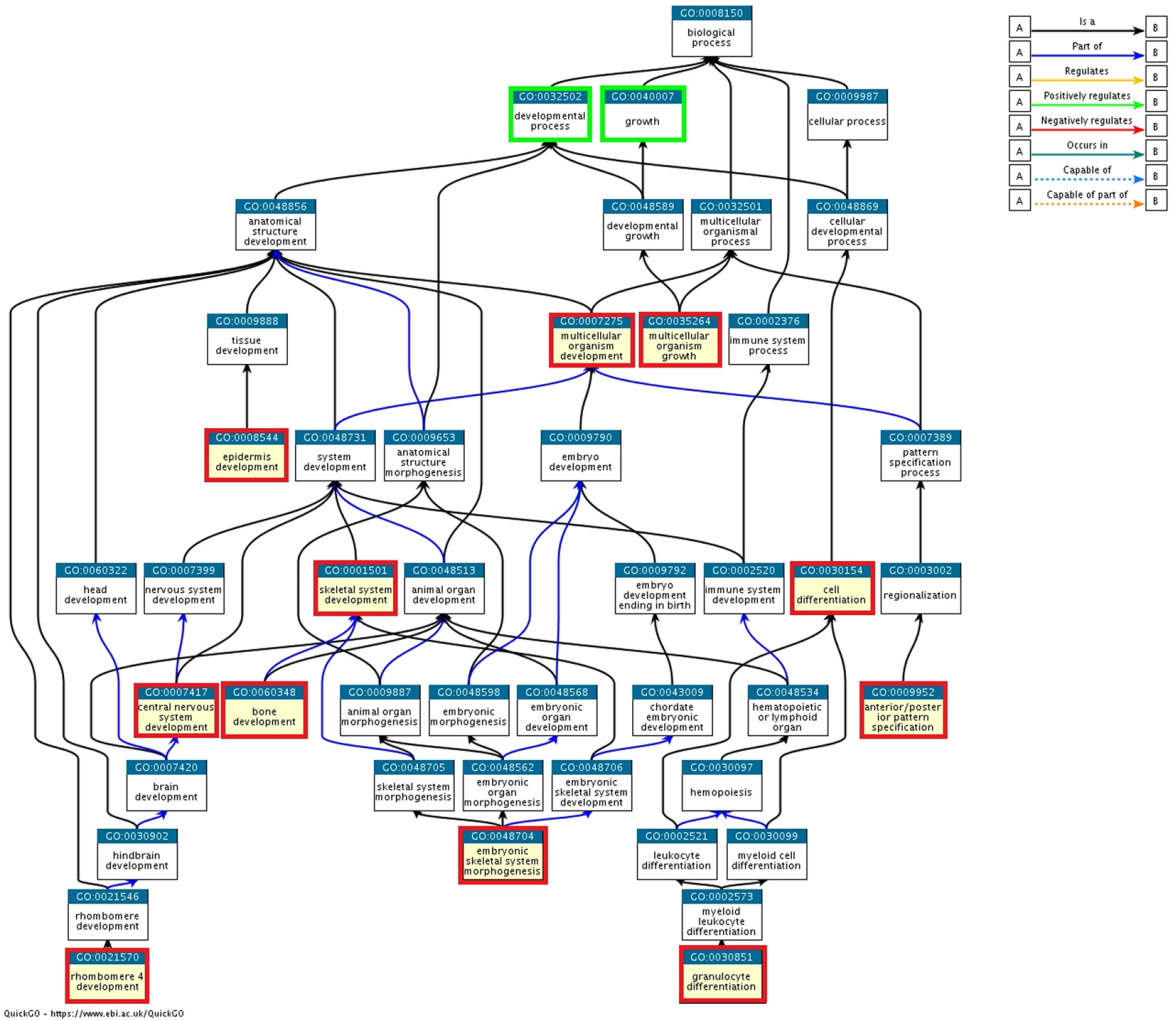
GO hierarchical structure for the eleven significantly enriched BPs (denoted with red color) associated with developmental process/growth term (denoted with green color). This GO tree was created and extracted by QuickGO^86^.

Four (module_ID: 2–5) out of the five modules exhibited enriched GO BPs while only three modules were associated with developmental processes according to QuickGO. Specifically, in module_2, a total number of 21 enriched GO BPs (Supplementary Table S4) and 78 participating genes were identified. According to QuickGO (see Figs 3 and 4), 9 out of the 21 GO BPs were related to development with 42 member genes (Supplementary Table S4). In the same module, 8 genes belonging to the homeobox B family genes along with *MDFI* were found to be enriched in embryonic skeletal system morphogenesis (GO:0048704). In module_3, none of the enriched GO BP terms were related to development (Supplementary Table S4). In module_4, three significantly enriched BPs (Supplementary Table S4) were identified in 7 member genes. Here, the only GO BP term that was associated with development through QuickGO was cell differentiation (GO:0030154) with 4 member genes (*PPARD, ELF2, ETV3L and ETV4*, Fig. 4). A total number of 29 GO BPs were found as significantly enriched in module_5 (Supplementary Table S4). Here, two development related processes i.e. multicellular organism growth (GO:0035264) and bone development (GO:0060348) were identified by QuickGO (Fig. 4) with 6 involved genes (*KAT2A, SP2, ANKRD11, RARA, BGLAP and AKAP13*).

### Functional candidate genes

An exhaustive list, including 66 modular genes, of the most plausible candidate genes for BW is provided in Table 3. From these genes, 52 were participating in enriched developmental processes, 7 were growth related genes that were not enriched to any developmental process and 7 were growth related genes identified in previous studies. These 66 modular genes were distributed across 7 chromosomes (GGA4, GGA10, GGA11, GGA15, GGA25, GGA26 and GGA27) with 47 of them detected in module_2. The *KRT (keratins)* family and B cluster of *HOX (homeobox)* family genes were also included here.

**Table 3:** List of 66 most plausible candidate genes for BW according to the following criteria: modular genes participating in enriched developmental processes, growth related modular genes not significantly enriched to any developmental process and growth related modular genes reported in previous studies.

Criterion	Gene	Description	Module_ID	GGA
modular genes participating in enriched developmental processes	*BTG2*	*BTG anti-proliferation factor 2*	module_2	26
	*ZAR1*	*zygote arrest 1*	module_2	4
	*MEOX1*	*mesenchyme homeobox 1*	module_2	27
	*KRT14*	*keratin 14*	module_2	27
	*KRT15*	*keratin 15*	module_2	27
	*TXK*	*TXK tyrosine kinase*	module_2	4
	*CSF3*	*colony stimulating factor 3*	module_2	27
	*ACAN*	*aggrecan*	module_2	10
	*HOXB1*	*homeobox B1*	module_2	27
	*HOXB2*	*homeobox B2*	module_2	27
	*HOXB3*	*homeobox B3*	module_2	27
	*HOXB4*	*homeobox B4*	module_2	27
	*HOXB5*	*homeobox B5*	module_2	27
	*HOXB6*	*homeobox B6*	module_2	27
	*HOXB7*	*homeobox B7*	module_2	27
	*HOXB8*	*homeobox B8*	module_2	27
	*HOXB9*	*homeobox B9*	module_2	27
	*HOXB13*	*homeobox B13*	module_2	27
	*MDFI*	*MyoD family inhibitor*	module_2	26
	*NES*	*nestin*	module_2	25
	*TBX21*	*T-box 21*	module_2	27
	*IGFBP4*	*insulin like growth factor binding protein 4*	module_2	27
	*PRELP*	*proline and arginine rich end leucine rich repeat protein*	module_2	26
	*HAPLN2*	*hyaluronan and proteoglycan link protein 2*	module_2	25
	*HAPLN3*	*hyaluronan and proteoglycan link protein 3*	module_2	10
	*GABRA4*	*gamma-aminobutyric acid type A receptor alpha4 subunit*	module_2	4
	*BCAN*	*brevican*	module_2	25
	*NHLH1*	*nescient helix-loop-helix 1*	module_2	25
	*ZBTB7B*	*zinc finger and BTB domain containing 7B*	module_2	25
	*FZD10*	*frizzled class receptor 10*	module_2	15
	*TCP11*	*t-complex 11*	module_2	26
	*PIWIL1*	*piwi like RNA-mediated gene silencing 1*	module_2	15
	*SPDEF*	*SAM pointed domain containing ETS transcription factor*	module_2	26
	*ZFPM1*	*zinc finger protein, FOG family member 1*	module_2	11
	*CBFA2T3*	*CBFA2/RUNX1 translocation partner 3*	module_2	11
	*KRT17*	*keratin 17*	module_2	27
	*CRABP2*	*cellular retinoic acid binding protein 2*	module_2	25
	*SH2D2A*	*SH2 domain containing 2 A*	module_2	25
	*NR1D1*	*nuclear receptor subfamily 1 group D member 1*	module_2	27
	*STX2*	*syntaxin 2*	module_2	15
	*TEC*	*tec protein tyrosine kinase*	module_2	4
	*ETV3*	*ETS variant 3*	module_2	25
	*PPARD*	*peroxisome proliferator activated receptor delta*	module_4	26
	*ELF2*	*E74 like ETS transcription factor 2*	module_4	4
	*ETV3L*	*ETS variant 3 like*	module_4	25
	*ETV4*	*ETS variant 4*	module_4	27
	*KAT2A*	*lysine acetyltransferase 2A*	module_5	27
	*RARA*	*retinoic acid receptor alpha*	module_5	27
	*BGLAP*	*bone gamma-carboxyglutamate protein*	module_5	25
	*SP2*	*Sp2 transcription factor*	module_5	27
	*ANKRD11*	*ankyrin repeat domain 11*	module_5	11
	*AKAP13*	*A-kinase anchoring protein 13*	module_5	10
growth related modular genes not significantly enriched to any developmental process	*GABRG1*	*gamma-aminobutyric acid type A receptor gamma1 subunit*	module_2	4
	*NGF*	*nerve growth factor*	module_2	26
	*APOBEC2*	*apolipoprotein B mRNA editing enzyme catalytic subunit 2*	module_5	26
	*STAT5B*	*signal transducer and activator of transcription 5B*	module_3	27
	*STAT3*	*signal transducer and activator of transcription 3*	module_5	27
	*SMAD4*	*SMAD family member 4*	module_5	25
	*MED1*	*mediator complex subunit 1*	module_5	27
growth related modular genes reported in previous studies	*CACNB1*	*calcium voltage-gated channel auxiliary subunit beta 1*	module_2	27
	*SLAIN2*	*SLAIN motif family member 2*	module_5	4
	*LEMD2*	*LEM domain containing 2*	module_5	26
	*ZC3H18*	*zinc finger CCCH-type containing 18*	module_5	11
	*TMEM132D*	*transmembrane protein 132D*	module_2	15
	*FRYL*	*FRY like transcription coactivator*	module_4	4
	*SGCB*	*sarcoglycan beta*	module_2	4

## Discussion

Results of the present study have shown that a typical quantitative trait such as that examined here is associated with modular genes exhibiting functional relevance to developmental processes. This means that application of functional enrichment analysis on modular genes can facilitate the identification of true causative genes for the trait under study. Following this approach, a total number of 52 functional candidate genes could be identified in the present study. Example genes that fall in this category were the following: *BTG2, ZAR1, MEOX1, KRT14, KRT15, TXK, CSF3, ACAN, HOXB, MDFI, NES, IGFBP4, PRELP, PPARD, ELF2, KAT2A, RARA and BGLAP*. Specifically, *BTG2, ZAR1, MEOX1, KRT14* and *KRT15* have been reported to participate in cerebellar development^30^, development of follicular oocytes^31^, somite differentiation^32^, keratinocytes proliferation^33^ and pigmentation of muscle tissues^34^, in chickens, respectively. *TXK (TXK tyrosine kinase)* has been reported as BW related gene^35^ while *CSF3 (colony stimulating factor 3)* has been described as a myelomonocytic growth factor in the species^36^. *ACAN (aggrecan)* is essential for cartilage formation during development in chicken and mouse mutants^37^ and the *HOX B* cluster genes are expressed in chick embryonic development^38^. The *MDFI* (MyoD family inhibitor) tumor suppressor gene is known to have a negative effect on myogenic regulatory factors^39^ while *NES (nestin)* is known as a neural progenitor cell marker during central nervous system development and a marker protein for neovascularization^40^. Furthermore, *IGFBP4 (insulin like growth factor binding protein 4)* is required for the adipose tissue development^41^ while *PRELP (proline and arginine rich end leucine rich repeat protein)* is highly expressed in cartilage, basement membranes, and bone development^42^. *PPARD (peroxisome proliferator activated receptor delta)* is a critical gene for normal adipose development and lipid homeostasis^43^ while *ELF2 (E74 like ETS transcription factor 2)* plays a key role in the development of lymphocytes^44^. *KAT2A (lysine acetyltransferase 2A)* is necessary for growth and differentiation of craniofacial cartilage and bone in zebrafish and mice^45^, *RARA (retinoic acid receptor alpha)* affects the hippocampal development^46^ and finally *BGLAP* is produced by osteoblasts shaping new bones in chickens^47^.

However, the search for modular genes that are exclusively enriched in functionally relevant terms has not proved to be efficient in identifying all true functional candidate genes. This finding may be fairly supported by the fact that 7 more genes (*GABRG1, NGF, APOBEC2, STAT5B, STAT3, SMAD4 and MED1*) that despite having well documented relevance to development were found to be enriched in other but developmental GO BP terms. Specifically, *GABRG1(gamma-aminobutyric acid type A receptor gamma1 subunit)* is reported as a BW related gene^35^ and *NGF (nerve growth factor)* is a regulator of the somite survival and axial rotation during early chicken embryo development^48^. *APOBEC2 (apolipoprotein B mRNA editing enzyme catalytic subunit 2)* is known as a critical regulator and maintainer of muscle development in mammals and might affect muscle development in chickens^49^. In the species, *STAT5B (signal transducer and activator of transcription 5B)* is associated with growth^50^. *STAT3 (signal transducer and activator of transcription 3)* plays a central role in development^51^, *SMAD4 (SMAD family member 4)* is a central mediator of the transforming growth factor β signaling pathway which affects among others the cell growth^52^ and finally *MED1 (mediator complex subunit 1)* has a key role in mammary epithelial cell growth^53^.

The list with the most plausible candidate genes for the trait was, however, not exhausted in the previous two categories since 7 more genes (*CACNB1, SLAIN2, LEMD2, ZC3H18, TMEM132D, FRYL and SGCB)* with well documented implication to BW, were completely omitted in any enrichment analysis. Most interestingly, five of the above genes (*CACNB1, SLAIN2, LEMD2, ZC3H18* and *TMEM132D)* contained lead SNPs. *CACNB1 (calcium voltage-gated channel auxiliary subunit beta 1)* has been reported to affect skeletal muscle development^54^ in mice. *SLAIN2 (SLAIN motif family member 2)* is necessary for the normal structure of microtubule cytoskeleton as it controls the microtubule growth during interphase^55^. *LEMD2 (LEM domain containing 2*) participates in nuclear structure organization^56^ and plays an important role in mouse embryonic development by regulating various signaling pathways such as MAPK (mitogen-activated protein kinase) and AKT (also known as Protein Kinase B)^57^. *ZC3H18 (zinc finger CCCH-type containing 18)* participates in RNA degradation^58^ and affects mRNA metabolism^59^. Finally, *TMEM132D (transmembrane protein 132D*) may function as a tumor suppressor gene^60^. Finally, both *FRYL (FRY like transcription coactivator)* and *SGCB (sarcoglycan beta)* have been associated with growth^61,62^ in the species.

The two aforementioned gene lists underline the potential limitations of a cluster based method such as that used here to assess the biological properties of the candidate gene sets. Specifically, these limitations relate to i) grouping of similar terms into a cluster and evaluating the enrichment of functional clusters instead of each individual term within the clusters and ii) the evaluation of the identified term clusters separately, while not taken into consideration the relationships between clusters^63^.

Apart from functional enrichment analysis, other analyses such as pathway analysis, gene network analysis and GBA gene prioritization analysis could also assist in identifying true causative genetic variants for the trait under study. For instance, in a previous study^64^, the use of GBA gene prioritization analysis on 1,012 positional candidate genes revealed 248 functional candidate genes for the same trait. However, fixed genomic regions (of 1 Mb) around the lead genomic markers were used in that study. A final interesting result of the present study was the discovery of 15 microRNAs within the 645 candidate genes for the trait under investigation. One of these, i.e. *MIR10A* has been reported as significant for feed intake in broilers^65^. *MIR10A* together with *MIR10B* have been reported to inhibit the development of human, mouse and rat granulosa cells during folliculogenesis^66^. Finally, *MIR7-2* has been reported as genomic locus for peroxisome proliferator activated receptor regulation^67^ and may have a functional role in hepatic lipid homeostasis. MicroRNAs have emerged as important regulators of gene expression post-transcriptionally and in *Gallus gallus* are known to play crucial roles in various biological processes such as the accumulation of abdominal fat^68^ and the lipid metabolism^69^.

In conclusion, the present GWAS revealed a large number of genomic regions and genes implicated in the genetic architecture of a complex trait such as the BW that fully complies with the Fisher’s infinitesimal model of inheritance. Exploitation of both community structure and functional enrichment analyses highlighted 3 modules as related to development. Current findings also indicated 52 modular genes participating in developmental processes and 14 more modular genes related to BW. Finally, the present study proposed 66 functional candidate genes for BW, some of which are novel and some identified candidates in previous studies.

## Methods

### Ethics statement

All animals included in this study were not subjected to any invasive procedures.

### Data and quality control

In total, n = 6,727 broilers (n = 3,735 males and n = 2,992 females) from a grand-grandparent (GGP) commercial line with records on BW at 35 days of age were made available by Aviagen Ltd. Phenotypic records for BW ranged from 1,130 to 2,630 g with an average of 1840.2 g (SD = 194 g). Animals were genotyped using the 600k Affymetrix® Axiom® high density genotyping array^4^ resulting in a total number of 578,815 SNPs. Only autosomal SNPs (n = 547,705) were considered. Quality control was performed first at a sample and second at a marker level. At a sample level, 72 females and 57 males were excluded due to call rate <0.99 and autosomal heterozygosity outside the 1.5 IQR (inter-quartile range) resulting in a number of n = 6,598 samples. At a marker level, a number of 285,717 SNPs were excluded due to: call rate <0.99, MAF (minor allele frequency) <0.01 and linkage disequilibrium (LD) r^2^ values greater than 0.99 within windows of 1 Mb inter-marker distance(s). A total of 6,598 samples and 262,067 SNPs were retained for GWAS. Quality control was performed using the SNP & Variation Suite software (version 8.8.1) of Golden Helix (http://www.goldenhelix.com).

### Statistical analysis

A multi-locus mixed-model (MLMM) stepwise regression with forward inclusion and backward elimination^70^ of SNPs was employed to identify genome-wide significant markers associated with the trait. The following statistical model was applied to the data:

$$y=X{\boldsymbol{\beta }}+w\alpha +{\rm Z}{\bf{u}}+e$$

where *y* is the n x 1 vector of phenotypic values of BW for n broilers, *X* is the n x 55 matrix of fixed effects: sex (2 classes), hatch (36 classes) and mating group (17 classes), ***β*** is the 55 × 1 vector of corresponding coefficients of fixed effects, ***w*** is the vector with elements of 0, 1, and 2 for the homozygote of the minor allele, heterozygote, and homozygote of the major allele, ***α*** is the vector of the fixed effect for the minor allele of the candidate SNP to be tested for association, *Z* is the incidence matrix relating observations to the polygenic random effects, ***u*** is the vector of polygenic random effects and ***e*** is the vector of random residuals.

The random effects were assumed to be normally distributed with zero means and the following covariance structure:

$$Var[\begin{array}{c}u\\ e\end{array}]=[\begin{array}{cc}G{\sigma }_{u}^{2} & 0\\ 0 & I{\sigma }_{e}^{2}\end{array}]$$

where ${\sigma }_{u}^{2}$ and ${\sigma }_{e}^{2}$ are the polygenic and error variance components, I is the nxn identity matrix, and G is the nxn genomic relationship matrix (GRM^71^) with elements of pairwise relationship coefficient using the 262,067 SNPs. Τhe genomic relationship coefficient between two individuals j and k, was estimated as follows:

$$\frac{1}{262,067}\sum _{i=1}^{262,067}\frac{(xij-2pi)(xik-2pi)}{2pi(1-2pi)}$$

where x_ij_ and x_ik_ represent the number (0, 1, or 2) of the minor allele for the i_th_ SNP of the j_th_ and k_th_ individuals, and p_i_ is the frequency of the minor allele^71^.

Statistically significant markers were selected at the optimal step of the MLMM stepwise regression according to extended Bayesian Information Criterion (eBIC^72^). P-values of these SNPs were then corrected for multiple comparisons using the false-discovery rate (FDR^73^) correction method. Here, a cut-off FDR p-value less than 0.05^74^ was considered as significant. The FDR p-value of 0.05 states that, among all observed results, 5% would be false positives.

A Quantile-quantile (Q-Q) plot was also used to analyze the extent to which the observed distribution of the test statistic followed the expected (null) distribution. This plot along with the estimation of the genomic inflation factor (λ) was done to assess potential systematic bias due to population structure or to the analytical approach^28^. This analysis was performed using the SNP & Variation Suite (version 8.8.1) software (Golden Helix: http://www.goldenhelix.com).

### Detection of candidate genomic regions with strong LD

We first estimated LD levels around each lead i.e. significant SNP. We then searched for genomic regions with strong LD around the lead SNPs defined as the maximum distance between the lead and the last SNP with D′ ≥ 0.8^75^. Note that, the D′, instead of the r^2^ LD measurement, was preferably used here as the first one is reported to be independent^76^ or less dependent^77^ on MAF. All LD calculations were performed using the SNP & Variation Suite (version 8.8.1) software (Golden Helix: http://www.goldenhelix.com).

### Identification of reported QTL and positional candidate genes

Next, we searched for growth/fatness related QTL in the ChickenQTLdb^1^ and positional candidate genes in the NCBI database^78,79^, within the strong LD genomic regions. Positions of QTL were remapped from *Gallus gallus 4* to *Gallus _gallus-5.0* assembly using the Genome Remapping Service from NCBI database^80^.

### Detection of community structure and functional module characterization

A gene network using all positional candidate genes was first constructed integrating the available *Homo sapiens* genes database (updated 17/3/2017) via the GeneMANIA V.3.4.1 plug-in^11^ in Cytoscape V3.6.0 (http://cytoscape.org/ ^81^). The gene network was built according to 7 types of interaction terms i.e. co-expression, co-localization, genetic interaction, pathway, physical interaction, predicted and shared protein domains. The automatic weighting method for network construction was also used while the number of related genes was set to zero.

Detection of community structure i.e. the appearance of densely interconnected nodes (modules) was then performed using the Girvan and Newman’s clustering algorithm^29^ via the GLay^82^ of clusterMaker^83^ plugin in Cytoscape^81^. This algorithm identifies modules within networks by repetitively removing edges with the highest “betweeness” i.e. edges between modules with higher values of betweeness rather than edges within modules. The strength of the network division into modules was also quantified using the modularity measure^29^. Typically, modularity values ranging from 0.3 to 0.7 are indicative of strong community structure^29^.

Modular genes were then subjected to GO Biological Process (BP) term enrichment analysis using the DAVID functional annotation tool (https://david.ncifcrf.gov/, version 6.8)^84^. The *Homo sapiens* species was also selected for the input gene list and as whole genome background for enrichment analysis. The following settings were used during this analysis: an EASE score (a modified Fisher exact p-value^85^) cut-off = 0.05 and a minimum number of genes per GO BP term = 2. GO biological processes with p-values lower than 0.05 were considered as significantly enriched. The QuickGO^86^ web-based tool was subsequently used to examine each resulting significantly enriched GO BP through browsing the hierarchical structure in the GO annotation database. GO BPs associated with developmental process or growth parent term(s) were considered as functionally relevant to the trait under study.

## Supplementary information

Table S1

Table S2

Table S3

Table S4

## Data Availability

The data that support the findings of this study are available from Aviagen Ltd. but restrictions apply to the availability of these data, which were used under license for the current study, and so are not publicly available. Data are however available from the authors upon reasonable request and with permission of Aviagen Ltd.
