# Supplementary material for: Discovery and characterization of functional modules associated with body weight in broilers
Source: Sci Rep. 2019 Jun 24;9:9125. doi: 10.1038/s41598-019-45520-5 (PMC6591351; doi:10.1038/s41598-019-45520-5)
Supplement: Supplementary file 1 — Table S1 [file 41598_2019_45520_MOESM1_ESM.pdf]

Discovery and characterization of functional modules associated with body weight in broilers

Eirini Tarsani[1*], Andreas Kranis[2,3], Gerasimos Maniatis[2], Santiago Avendano[2], Ariadne L. Hager-Theodorides[1], Antonios Kominakis[1]

[1]Department of Animal Science and Aquaculture, Agricultural University of Athens, Iera Odos 75, 11855, Athens, Greece

[2]Aviagen Ltd., Newbridge, Midlothian EH28 8SZ, UK

[3] The Roslin Institute, University of Edinburgh, EH25 9RG, Midlothian, United Kingdom

*corresponding author: etarsani@aua.gr

Table S1: Positional candidate genes for BW.

**SNP ID**	**GGA**	**Position of the marker (bp)**	***Gene ID****	***Description***	**Start position of the gene (bp)***	**End position of the gene (bp)***	**Orientation of the gene**	**Minimum distance from gene (bp)**
rs13923872	1	112,741,685	*USP9X*	*ubiquitin specific peptidase 9, X-linked*	112,078,329	112,176,727	minus	564,958
			LOC101750779	*uncharacterized LOC101750779*	112,193,534	112,198,521	plus	543,164
			LOC107056757	*uncharacterized LOC107056757*	112,223,451	112,228,082	minus	513,603
			MED14	*mediator complex subunit 14*	112,228,091	112,262,695	plus	478,99

			LOC107056765	*uncharacterized LOC107056765*	112,263,096	112,268,266	plus	473,419
			ATP6AP2	*ATPase H+ transporting accessory protein 2*	112,273,187	112,282,377	minus	459,308
			LOC101750924	*uncharacterized LOC101750924*	112,282,457	112,304,013	plus	437,672
			LOC107056538	*basic proline-rich protein-like*	112,349,429	112,354,018	minus	387,667
			BCOR	*BCL6 corepressor*	112,378,844	112,422,324	plus	319,361
			LOC107057081	*uncharacterized LOC107057081*	112,474,240	112,486,871	minus	254,814
			LOC101751364	*uncharacterized LOC101751364*	112,482,075	112,497,856	plus	243,829
			LOC107057076	*uncharacterized LOC107057076*	112,498,548	112,504,661	plus	237,024
			LOC101751443	*uncharacterized LOC101751443*	112,519,737	112,523,119	plus	218,566
			MIR6672	*microRNA 6672*	112,625,566	112,625,675	plus	116,01
			LOC107057061	*uncharacterized LOC107057061*	112,635,402	112,643,712	plus	97,973
			LOC107057049	*uncharacterized LOC107057049*	112,668,313	112,707,049	minus	34,636
			MID1IP1	*MID1 interacting protein 1*	112,738,605	112,739,753	minus	1,932
			TSPAN7	*tetraspanin 7*	112,766,324	112,855,838	minus	24,639
			LOC101751659	*uncharacterized LOC101751659*	112,767,424	112,769,932	plus	25,739
			LOC107057044	*uncharacterized LOC107057044*	112,853,920	112,855,360	minus	112,235
			OTC	*ornithine carbamoyltransferase*	112,898,625	112,924,531	minus	156,94
			RPGR	*retinitis pigmentosa GTPase regulator*	112,926,169	112,985,737	plus	184,484
			SRPX	*sushi repeat containing protein, X-linked*	112,994,814	113,035,754	plus	253,129
			SYTL5	*synaptotagmin like 5*	113,039,522	113,117,936	minus	297,837
			DYNLT3	*dynein light chain Tctex-type 3*	113,133,105	113,139,987	plus	391,42
			CYBB	*cytochrome b-245 beta chain*	113,149,626	113,183,626	minus	407,941
			XK	*X-linked Kx blood group*	113,199,116	113,214,148	minus	457,431
			LANCL3	*LanC like 3*	113,215,605	113,251,683	minus	473,92

			LOC107056992	*uncharacterized LOC107056992*	113,278,216	113,282,701	minus	536,531
			PRRG1	*proline rich and Gla domain 1*	113,283,937	113,314,070	minus	542,252
			LOC107056924	*maestro heat-like repeat-containing protein family member 2B*	113,325,651	113,339,179	plus	583,966
			LOC107056990	*maestro heat-like repeat-containing protein family member 2B*	113,343,513	113,348,692	plus	601,828
			LOC107056981	*maestro heat-like repeat-containing protein family member 2B*	113,350,799	113,354,007	plus	609,114
rs312691174	4	29,074,989	*LOC107051793*	*uncharacterized LOC107051793*	28,550,125	28,563,120	minus	511,869
			PCDH18	*protocadherin 18*	28,880,214	28,890,093	minus	184,896
			LOC107051792	*uncharacterized LOC107051792*	28,986,991	29,036,383	plus	38,606
			SLC7A11	*solute carrier family 7 member 11*	29,138,177	29,196,482	minus	63,188
			LOC101751121	*uncharacterized LOC101751121*	29,205,067	29,277,125	minus	130,078
			NOCT	*nocturnin*	29,428,771	29,434,135	plus	353,782
			ELF2	*E74 like ETS transcription factor 2*	29,438,718	29,467,618	minus	363,729
			MGARP	*mitochondria localized glutamic acid rich protein*	29,471,760	29,493,863	minus	396,771
			LOC107051797	*atherin-like*	29,494,724	29,497,114	minus	419,735
			NAA15	*N(alpha)-acetyltransferase 15, NatA auxiliary subunit*	29,496,798	29,534,035	plus	421,809
			RAB33B	*RAB33B, member RAS oncogene family*	29,539,318	29,546,334	plus	464,329
			LOC422442	*uncharacterized LOC422442*	29,559,136	29,561,953	minus	484,147
			SETD7	*SET domain containing lysine methyltransferase 7*	29,561,960	29,578,876	minus	486,971
			MGST2	*microsomal glutathione S-transferase 2*	29,593,387	29,603,029	plus	518,398
			LOC107051791	*microsomal glutathione S-transferase 2-like*	29,604,032	29,611,350	plus	529,043

			MAML3	*mastermind like transcriptional coactivator 3*	29,611,253	29,815,133	minus	536,264
rs15608447	4	66,885,210	*FIP1L1*	*factor interacting with PAPOLA and CPSF1*	66,133,445	66,171,020	minus	714,19
			SCFD2	*sec1 family domain containing 2*	66,173,796	66,368,297	plus	516,913
			LOC107053245	*uncharacterized LOC107053245*	66,257,009	66,266,179	minus	619,031
			RASL11B	*RAS like family 11 member B*	66,369,830	66,373,534	minus	511,676
			LOC107053244	*uncharacterized LOC107053244*	66,391,577	66,398,660	minus	486,55
			LOC422757	*uncharacterized LOC422757*	66,403,272	66,405,708	minus	479,502
			USP46	*ubiquitin specific peptidase 46*	66,413,295	66,440,521	plus	444,689
			SPATA18	*spermatogenesis associated 18*	66,567,846	66,587,444	minus	297,766
			SGCB	*sarcoglycan beta*	66,587,612	66,593,633	plus	291,577
			LRRC66	*leucine rich repeat containing 66*	66,593,723	66,602,814	plus	282,396
			DCUN1D4	*defective in cullin neddylation 1 domain containing 4*	66,607,031	66,645,141	minus	240,069
			CWH43	*cell wall biogenesis 43 C-terminal homolog*	66,651,068	66,677,010	minus	208,2
			OCIAD1	*OCIA domain containing 1*	66,693,766	66,708,277	minus	176,933
			FRYL	*FRY like transcription coactivator*	66,708,277	66,866,440	plus	18,77
			ZAR1	*zygote arrest 1*	66,868,239	66,871,111	minus	14,099
			LOC107053243	***uncharacterized LOC107053243***	66,870,041	66,885,878	plus	0
			SLC10A4	*solute carrier family 10 member 4*	66,870,984	66,872,626	minus	12,584
			SLAIN2	***SLAIN motif family member 2***	66,883,481	66,910,807	minus	0
			TEC	*tec protein tyrosine kinase*	66,928,003	66,969,829	plus	42,793
			TXK	*TXK tyrosine kinase*	66,970,277	66,990,459	plus	85,067
			NIPAL1	*NIPA like domain containing 1*	66,992,447	67,002,107	minus	107,237

			CNGA1	*cyclic nucleotide gated channel alpha 1*	67,007,806	67,015,296	plus	122,596
			NFXL1	*nuclear transcription factor, X-box binding like 1*	67,016,510	67,061,474	plus	131,3
			CORIN	*corin, serine peptidase*	67,063,312	67,186,506	plus	178,102
			ATP10D	*ATPase phospholipid transporting 10D (putative)*	67,189,360	67,228,010	minus	304,15
			COMMD8	*COMM domain containing 8*	67,239,252	67,243,040	plus	354,042
			LOC107053239	*uncharacterized LOC107053239*	67,246,293	67,253,895	minus	361,083
			LOC107053241	*uncharacterized LOC107053241*	67,252,999	67,266,059	plus	367,789
			GABRB1	*gamma-aminobutyric acid type A receptor beta1 subunit*	67,253,915	67,271,613	minus	368,705
			LOC107053242	*uncharacterized LOC107053242*	67,325,419	67,334,233	plus	440,209
			LOC107053240	*uncharacterized LOC107053240*	67,330,651	67,347,343	minus	445,441
			GABRA4	*gamma-aminobutyric acid type A receptor alpha4 subunit*	67,355,338	67,402,602	plus	470,128
			GABRA2	*gamma-aminobutyric acid type A receptor alpha2 subunit*	67,487,682	67,549,247	plus	602,472
			LOC107053237	*uncharacterized LOC107053237*	67,489,979	67,491,477	minus	604,769
			LOC107053238	*uncharacterized LOC107053238*	67,546,207	67,576,158	minus	660,997
			GABRG1	*gamma-aminobutyric acid type A receptor gamma1 subunit*	67,576,299	67,633,176	plus	691,089
rs318199727	10	13,536,548	*LOC107054202*	*uncharacterized LOC107054202*	12,796,949	12,800,712	minus	735,836
			PEX11A	*peroxisomal biogenesis factor 11 alpha*	12,815,015	12,820,635	plus	715,913
			LOC107054203	*uncharacterized LOC107054203*	12,817,514	12,823,049	minus	713,499
			PLIN1	*perilipin 1*	12,822,263	12,826,843	plus	709,705
			KIF7	*kinesin family member 7*	12,827,027	12,836,867	plus	699,681

			TICRR	*TOPBP1 interacting checkpoint and replication regulator*	12,837,814	12,854,365	minus	682,183
			RHCG	*Rh family C glycoprotein*	12,859,333	12,867,258	plus	669,29
			LOC107054204	*uncharacterized LOC107054204*	12,902,048	12,938,774	minus	597,774
			TRNAR-UCG	*transfer RNA arginine (anticodon UCG)*	12,942,837	12,942,909	minus	593,639
			POLG	*DNA polymerase gamma, catalytic subunit*	12,942,988	12,953,027	plus	583,521
			FANCI	*Fanconi anemia complementation group I*	12,951,763	12,975,529	minus	561,019
			RLBP1	*retinaldehyde binding protein 1*	12,976,366	12,980,554	plus	555,994
			ABHD2	*abhydrolase domain containing 2*	12,981,973	13,021,732	minus	514,816
			MFGE8	*milk fat globule-EGF factor 8 protein*	13,033,323	13,040,620	plus	495,928
			HAPLN3	*hyaluronan and proteoglycan link protein 3*	13,042,935	13,046,872	plus	489,676
			ACAN	*aggrecan*	13,047,289	13,092,387	minus	444,161
			AEN	*apoptosis enhancing nuclease*	13,131,452	13,134,038	minus	402,51
			MIR1720	*microRNA 1720*	13,134,585	13,134,649	minus	401,899
			MIR7-2	*microRNA 7-2*	13,134,720	13,134,818	minus	401,73
			MIR3529	*microRNA 3529*	13,134,724	13,134,814	plus	401,734
			DET1	*de-etiolated homolog 1 (Arabidopsis)*	13,149,476	13,171,850	plus	364,698
			MRPS11	*mitochondrial ribosomal protein S11*	13,171,107	13,174,638	minus	361,91
			MRPL46	*mitochondrial ribosomal protein L46*	13,174,668	13,177,215	plus	359,333
			LOC101751754	*uncharacterized LOC101751754*	13,178,649	13,201,172	plus	335,376
			LOC101751792	*uncharacterized LOC101751792*	13,196,390	13,209,087	minus	327,461
			NTRK3	*neurotrophic receptor tyrosine kinase 3*	13,227,881	13,408,049	plus	128,499
			LOC107054207	*uncharacterized LOC107054207*	13,381,318	13,386,137	minus	150,411

			LOC107054206	***uncharacterized LOC107054206***	13,388,442	13,616,738	minus	0
			AGBL1	*ATP/GTP binding protein like 1*	13,623,118	13,914,436	minus	86,57
			KLHL25	*kelch like family member 25*	13,954,963	13,969,663	plus	418,415
			AKAP13	*A-kinase anchoring protein 13*	13,974,501	14,169,240	minus	437,953
			LOC107054188	*uncharacterized LOC107054188*	14,182,433	14,183,328	minus	645,885
			SV2B	*synaptic vesicle glycoprotein 2B*	14,187,010	14,235,623	plus	650,462
rs318098582	11	18,651,449	*BANP*	*BTG3 associated nuclear protein*	18,243,614	18,386,758	plus	264,691
			ZNF469	*zinc finger protein 469*	18,416,335	18,579,095	plus	72,354
			LOC107054329	*uncharacterized LOC107054329*	18,541,302	18,548,014	minus	103,435
			ZFPM1	*zinc finger protein, FOG family member 1*	18,584,457	18,613,764	plus	37,685
			CIDEC	*cell death inducing DFFA like effector c*	18,614,459	18,616,305	minus	35,144
			ZC3H18	***zinc finger CCCH-type containing 18***	18,617,357	18,656,718	plus	0
			MIR1571	*microRNA 1571*	18,632,364	18,632,461	plus	18,988
			IL17C	*interleukin 17C*	18,658,034	18,663,248	plus	6,585
			CYBA	*cytochrome b-245 alpha chain*	18,663,342	18,665,615	minus	11,893
			MVD	*mevalonate diphosphate decarboxylase*	18,665,712	18,668,663	minus	14,263
			RNF166	*ring finger protein 166*	18,670,083	18,677,685	minus	18,634
			CTU2	*cytosolic thiouridylase subunit 2*	18,677,742	18,681,201	plus	26,293
			PIEZO1	*piezo type mechanosensitive ion channel component 1*	18,681,139	18,700,645	minus	29,69
			LOC107054330	*nascent polypeptide-associated complex subunit alpha, muscle-specific form-like*	18,696,540	18,698,959	plus	45,091
			CDT1	*chromatin licensing and DNA replication factor 1*	18,701,940	18,705,724	plus	50,491

			APRT	*adenine phosphoribosyltransferase*	18,706,797	18,709,506	minus	55,348
			GALNS	*galactosamine (N-acetyl)-6-sulfatase*	18,714,200	18,758,480	minus	62,751
			TRAPPC2L	*trafficking protein particle complex 2 like*	18,758,475	18,761,079	plus	107,026
			PABPN1L	*poly(A) binding protein nuclear 1 like, cytoplasmic*	18,762,170	18,765,810	minus	110,721
			CBFA2T3	*CBFA2/RUNX1 translocation partner 3*	18,767,307	18,788,285	minus	115,858
			ACSF3	*acyl-CoA synthetase family member 3*	18,805,694	18,846,630	plus	154,245
			CDH15	*cadherin 15*	18,848,471	18,853,003	plus	197,022
			SLC22A31	*solute carrier family 22 member 31*	18,853,081	18,856,750	minus	201,632
			ANKRD11	*ankyrin repeat domain 11*	18,857,696	18,939,049	minus	206,247
			MIR1560	*microRNA 1560*	18,874,320	18,874,423	minus	222,871
			MIR1785	*microRNA 1785*	18,926,659	18,926,760	minus	275,21
			SPG7	*SPG7, paraplegin matrix AAA peptidase subunit*	18,946,166	18,976,689	plus	294,717
rs317945754	15	3,557,083	*EP400*	*E1A binding protein p400*	2,582,896	2,628,948	minus	928,135
			PUS1	*pseudouridylate synthase 1*	2,629,662	2,634,569	minus	922,514
			ULK1	*unc-51 like autophagy activating kinase 1*	2,635,904	2,708,526	minus	848,557
			MMP17	*matrix metallopeptidase 17*	2,722,077	2,765,544	minus	791,539
			SFSWAP	*splicing factor SWAP homolog*	2,792,492	2,832,182	minus	724,901
			STX2	*syntaxin 2*	2,926,591	3,223,484	plus	333,599
			ADGRD1	*adhesion G protein-coupled receptor D1*	3,062,231	3,191,688	minus	365,395
			RAN	*RAN, member RAS oncogene family*	3,201,356	3,205,602	minus	351,481
			RIMBP2	*RIMS binding protein 2*	3,223,574	3,347,096	plus	209,987

			PIWIL1	*piwi like RNA-mediated gene silencing 1*	3,347,780	3,421,290	minus	135,793
			FZD10	*frizzled class receptor 10*	3,432,876	3,435,118	minus	121,965
			LOC107051610	*frizzled-10-like*	3,442,998	3,446,063	minus	111,02
			TMEM132D	***transmembrane protein 132D***	3,526,053	3,718,886	plus	0
			GLT1D1	*glycosyltransferase 1 domain containing 1*	3,736,272	3,784,033	minus	179,189
			SLC15A4	*solute carrier family 15 member 4*	3,785,313	3,806,720	plus	228,23
			TMEM132C	*transmembrane protein 132C*	3,830,248	4,010,779	minus	273,165
			LOC107051578	*uncharacterized LOC107051578*	4,105,652	4,107,324	minus	548,569
			LOC107051609	*uncharacterized LOC107051609*	4,241,859	4,320,316	plus	684,776
			TMEM132B	*transmembrane protein 132B*	4,291,921	4,471,778	minus	734,838
			AACS	*acetoacetyl-CoA synthetase*	4,478,298	4,515,607	minus	921,215
rs316794400	22	4,594,855	*LOC101751469*	*uncharacterized LOC101751469*	4,576,077	4,577,398	minus	17,457
			ANXA4	*annexin A4*	4,584,810	4,594,055	plus	800
			SLC20A1	*solute carrier family 20 member 1*	4,596,702	4,604,387	plus	1,847
			NT5DC4	*5'-nucleotidase domain containing 4*	4,604,777	4,609,445	plus	9,922
			CKAP2L	*cytoskeleton associated protein 2 like*	4,609,435	4,616,294	minus	14,58
			LOC107054991	*uncharacterized LOC107054991*	4,616,423	4,616,974	plus	21,568
			IL1B	*interleukin 1, beta*	4,616,889	4,618,625	minus	22,034
rs317288536	25	976,833	*BGLAP*	*bone gamma-carboxyglutamate protein*	594	1,789	plus	975,044
			SMG5	*SMG5, nonsense mediated mRNA decay factor*	2,171	24,198	minus	952,635
			TMEM79	*transmembrane protein 79*	24,606	27,213	plus	949,62
			GLMP	*glycosylated lysosomal membrane protein*	27,661	31,956	minus	944,877

			CCT3	*chaperonin containing TCP1 subunit 3*	33,641	44,203	minus	932,63
			LOC107055078	*uncharacterized LOC107055078*	46,465	47,469	minus	929,364
			LOC107055080	*nectin-4-like*	54,112	60,599	minus	916,234
			LIM2	*lens intrinsic membrane protein 2*	61,134	68,96	minus	907,873
			LOC107055082	*cytochrome b5 domain-containing protein 1-like*	81,045	82,719	minus	894,114
			LOC107055083	*uncharacterized LOC107055083*	82,856	89,386	plus	887,447
			VPS45	*vacuolar protein sorting 45 homolog*	92,295	119,429	plus	857,404
			PLEKHO1	*pleckstrin homology domain containing O1*	123,481	139,888	plus	836,945
			LOC107055081	*uncharacterized LOC107055081*	142,235	150,69	plus	826,143
			ANP32E	*acidic nuclear phosphoprotein 32 family member E*	162,05	174,225	minus	802,608
			LOC100859767	*cytochrome b5 domain-containing protein 1-like*	204	205,667	plus	771,166
			APOA1BP	*apolipoprotein A-I binding protein*	224,358	227,444	plus	749,389
			GPATCH4	*G-patch domain containing 4*	227,411	231,838	minus	744,995
			LOC107055084	*uncharacterized LOC107055084*	235,211	236,685	plus	740,148
			MEX3A	*mex-3 RNA binding family member A*	247,371	260,425	minus	716,408
			LOC107055086	*sperm-associated antigen 4 protein-like*	783,429	786,252	minus	190,581
			LOC100857131	*sperm-associated antigen 4 protein-like*	797,89	798,435	minus	178,398
			UBQLN4	*ubiquilin 4*	804,221	815,017	minus	161,816
			LAMTOR2	*late endosomal/lysosomal adaptor, MAPK and MTOR activator 2*	815,073	817,92	plus	158,913
			RAB25	*RAB25, member RAS oncogene family*	818,004	824,803	plus	152,03

				RAB2B	*RAB2B, member RAS oncogene family*	825,467	830,852	plus	145,981
				LOC101747704	*uncharacterized LOC101747704*	846,955	849,648	minus	127,185
				LOC107055087	*sperm-associated antigen 4 protein-like*	855,482	858,864	minus	117,969
				OTUD7B	*OTU deubiquitinase 7B*	888,239	923,782	plus	53,051
				MTMR11	*myotubularin related protein 11*	925,626	933,506	plus	43,327
				SF3B4	*splicing factor 3b subunit 4*	933,59	938,879	plus	37,954
				SV2A	*synaptic vesicle glycoprotein 2A*	939,052	949,843	plus	26,99
				LOC107055093	*uncharacterized LOC107055093*	950,637	951,127	plus	25,706
				LOC107055108	*feather keratin 3-like*	953,49	954,648	plus	22,185
				LOC107055109	*feather keratin 3-like*	956,426	957,314	plus	19,519
				LOC100859249	*feather keratin 3-like*	959,38	960,434	plus	16,399
				LOC107055107	*feather keratin 1-like*	959,446	963,95	plus	12,883
				LOC100859427	*feather keratin 1-like*	966,46	967,631	plus	9,202
				LOC426914	*feather keratin 1-like*	969,985	971,047	plus	5,786
				F-KER	***feather keratin I***	973,471	980,575	plus	0
				LOC429492	*keratin D*	973,475	974,554	plus	2,279
				LOC431325	***feather keratin 1-like***	976,657	980,857	plus	0
				LOC431324	*keratin A*	979,736	983,908	plus	2,903
				LOC426913	*feather keratin 1-like*	982,753	987,425	plus	5,92
				LOC431323	*beta-keratin-related protein-like*	991,47	993,152	plus	14,637
				LOC431322	*feather keratin 1-like*	997,064	997,414	plus	20,231
				LOC431321	*keratin*	1,002,033	1,004,112	plus	25,2
				LOC431320	*feather beta keratin-like*	1,008,291	1,010,072	plus	31,458
				LOC107055103	*scale keratin-like*	1,010,842	1,012,163	minus	34,009

			LOC107055106	*uncharacterized LOC107055106*	1,014,345	1,015,145	plus	37,512
			LOC431317	*scale keratin-like*	1,017,251	1,017,835	minus	40,418
			LOC431316	*scale keratin-like*	1,018,813	1,019,553	plus	41,98
			LOC100859586	*scale keratin-like*	1,020,735	1,021,650	minus	43,902
			LOC100859657	*scale keratin-like*	1,020,928	1,025,554	minus	44,095
			LOC100859616	*scale keratin-like*	1,022,275	1,023,023	plus	45,442
			LOC425362	*scale keratin-like*	1,026,144	1,027,006	plus	49,311
			LOC100857270	*scale keratin-like*	1,028,628	1,029,557	minus	51,795
			LOC100859756	*scale keratin-like*	1,030,236	1,034,932	plus	53,403
			LOC100859722	*scale keratin-like*	1,030,356	1,031,017	plus	53,523
			LOC100857297	*scale keratin-like*	1,032,585	1,044,971	minus	55,752
			LOC107055105	*uncharacterized LOC107055105*	1,032,750	1,040,322	plus	55,917
			LOC426912	*scale keratin-like*	1,036,532	1,037,365	minus	59,699
			LOC100859790	*scale keratin-like*	1,037,906	1,038,601	plus	61,073
			LOC101751554	*scale keratin-like*	1,040,076	1,040,849	minus	63,243
			LOC100857367	*scale keratin-like*	1,041,747	1,042,135	plus	64,914
			LOC107055104	*scale keratin-like*	1,044,261	1,044,828	minus	67,428
			LOC101750668	*scale keratin-like*	1,045,428	1,046,372	plus	68,595
			LOC396480	*keratin*	1,048,622	1,050,613	minus	71,789
			LOC101750550	*scale keratin-like*	1,052,489	1,053,802	plus	75,656
			LOC396479	*keratin*	1,055,266	1,056,859	minus	78,433
			LOC431314	*scale keratin-like*	1,058,333	1,060,625	plus	81,5
			LOC769486	*scale keratin-like*	1,064,610	1,066,554	plus	87,777
			LOC107055102	*uncharacterized LOC107055102*	1,067,878	1,069,936	plus	91,045
			LOC408038	*beta-keratin*	1,069,829	1,071,422	minus	92,996

			LOC431313	*feather beta keratin-like*	1,074,635	1,075,569	plus	97,802
			LOC107055092	*uncharacterized LOC107055092*	1,078,270	1,079,527	minus	101,437
			LOC100857468	*feather keratin Cos1-1/Cos1-3/Cos2-1-like*	1,080,919	1,087,221	minus	104,086
			LOC107055101	*uncharacterized LOC107055101*	1,093,075	1,095,264	minus	116,242
			LOC101751614	*keratin, type I cytoskeletal 9-like*	1,098,677	1,100,340	minus	121,844
			LOC107055091	*beta-keratin-related protein-like*	1,102,072	1,103,363	plus	125,239
			LOC107055090	*uncharacterized LOC107055090*	1,106,254	1,107,828	plus	129,421
			LOC107055100	*uncharacterized LOC107055100*	1,109,328	1,111,524	minus	132,495
			LOC101751113	*titin-like*	1,116,120	1,122,481	plus	139,287
			LOC107055099	*uncharacterized LOC107055099*	1,116,136	1,119,078	minus	139,303
			EDYM2	*epidermal differentiation protein containing Y motif 2*	1,122,288	1,125,199	minus	145,455
			EDQREP	*epidermal differentiation protein containing glutamine (Q) repeats*	1,127,324	1,130,658	minus	150,491
			EDPE	*epidermal differentiation protein rich in proline and glutamic acid (E)*	1,139,306	1,142,301	plus	162,473
			LOC107055098	*epidermal differentiation protein containing glutamine (Q) repeats-like*	1,144,136	1,145,825	minus	167,303
			EDQCM	*epidermal differentiation protein containing QC motifs*	1,148,589	1,150,441	minus	171,756
			EDDM	*epidermal differentiation protein containing DPCC motifs*	1,154,359	1,157,611	minus	177,526
			EDNC	*epidermal differentiation protein encoded by neighbor of cornulin*	1,159,110	1,161,256	plus	182,277
			CRNN	*cornulin*	1,168,202	1,170,459	plus	191,369
			SCFN	*scaffoldin*	1,173,597	1,177,833	plus	196,764
			LOC107055094	*trichohyalin-like*	1,178,020	1,190,574	plus	201,187

				S100A11	*S100 calcium binding protein A11*	1,201,165	1,202,884	plus	224,332
				COPA	*coatomer protein complex subunit alpha*	1,203,325	1,221,735	minus	226,492
				NCSTN	*nicastrin*	1,221,813	1,232,683	plus	244,98
				NHLH1	*nescient helix-loop-helix 1*	1,241,431	1,246,232	plus	264,598
				LOC107055095	*uncharacterized LOC107055095*	1,246,142	1,253,267	minus	269,309
				VANGL2	*VANGL planar cell polarity protein 2*	1,250,127	1,262,898	plus	273,294
				LY9	*lymphocyte antigen 9*	1,266,694	1,272,789	minus	289,861
				SLAMF1	*signaling lymphocytic activation molecule family member 1*	1,274,631	1,280,327	minus	297,798
				CD48	*CD48 molecule*	1,282,479	1,284,960	minus	305,646
				CD244	*CD244 molecule*	1,285,835	1,293,468	minus	309,002
				LOC101750757	*uncharacterized LOC101750757*	1,296,055	1,297,931	plus	319,222
				KIRREL	*kin of IRRE like (Drosophila)*	1,301,854	1,321,563	plus	325,021
				LOC101750908	*T-lymphocyte surface antigen Ly-9-like*	1,323,643	1,330,948	plus	346,81
				SLAMF8	*SLAM family member 8*	1,331,041	1,334,671	plus	354,208
				ETV3	*ETS variant 3*	1,334,908	1,344,750	plus	358,075
				ETV3L	*ETS variant 3 like*	1,353,678	1,355,999	plus	376,845
				ARHGEF11	*Rho guanine nucleotide exchange factor 11*	1,357,481	1,378,565	plus	380,648
				LRRC71	*leucine rich repeat containing 71*	1,379,498	1,382,513	minus	402,665
				PEAR1	*platelet endothelial aggregation receptor 1*	1,382,768	1,391,921	minus	405,935
				NTRK1	*neurotrophic receptor tyrosine kinase 1*	1,394,975	1,402,575	minus	418,142
				INSRR	*insulin receptor related receptor*	1,403,569	1,413,068	minus	426,736
				LOC100857512	*death-associated protein kinase 2-like*	1,413,151	1,416,561	minus	436,318

			SH2D2A	*SH2 domain containing 2A*	1,416,567	1,421,255	plus	439,734
			PRCC	*papillary renal cell carcinoma (translocation-associated)*	1,421,164	1,430,083	minus	444,331
			HDGF	*hepatoma-derived growth factor*	1,432,110	1,437,580	plus	455,277
			MRPL24	*mitochondrial ribosomal protein L24*	1,438,014	1,439,195	plus	461,181
			RRNAD1	*ribosomal RNA adenine dimethylase domain containing 1*	1,439,293	1,442,440	minus	462,46
			CRABP2	*cellular retinoic acid binding protein 2*	1,448,446	1,451,746	plus	471,613
			LOC425431	*dnaJ homolog subfamily A member 1-like*	1,455,339	1,458,566	minus	478,506
			NES	*nestin*	1,462,876	1,470,387	minus	486,043
			BCAN	*brevican*	1,473,720	1,486,466	plus	496,887
			HAPLN2	*hyaluronan and proteoglycan link protein 2*	1,487,083	1,489,801	minus	510,25
			RHBG	*Rh family B glycoprotein*	1,490,958	1,496,016	plus	514,125
			LOC107055112	*uncharacterized LOC107055112*	1,505,796	1,508,625	minus	528,963
			LOC107055111	*uncharacterized LOC107055111*	1,521,253	1,544,127	minus	544,42
			MEF2D	*myocyte enhancer factor 2D*	1,557,855	1,582,946	minus	581,022
			LOC107055110	*uncharacterized LOC107055110*	1,598,470	1,599,898	plus	621,637
			LOC101750487	*uncharacterized LOC101750487*	1,609,167	1,620,210	minus	632,334
			LOC101750716	*uncharacterized LOC101750716*	1,620,907	1,627,137	minus	644,074
			LOC107055114	*E3 SUMO-protein ligase PIAS3-like*	1,636,879	1,651,430	plus	660,046
			MIR6662	*microRNA 6662*	1,637,951	1,638,060	minus	661,118
			INTS3	*integrator complex subunit 3*	1,654,016	1,685,697	minus	677,183
			LOC107055115	*atrial natriuretic peptide receptor 1-like*	1,686,320	1,696,310	minus	709,487

			LOC107055116	*atrial natriuretic peptide receptor 1-like*	1,696,623	1,700,257	minus	719,79
			ILF2	*interleukin enhancer binding factor 2*	1,701,006	1,705,564	plus	724,173
			SNAPIN	*SNAP associated protein*	1,705,771	1,706,895	minus	728,938
			IL6R	*interleukin 6 receptor*	1,708,497	1,714,085	plus	731,664
			SHE	*Src homology 2 domain containing E*	1,715,301	1,720,734	minus	738,468
			UBE2Q1	*ubiquitin conjugating enzyme E2 Q1*	1,722,132	1,729,019	minus	745,299
			CHRNB2	*cholinergic receptor nicotinic beta 2 subunit*	1,729,683	1,734,479	plus	752,85
			ADAR	*adenosine deaminase, RNA specific*	1,735,473	1,747,289	minus	758,64
			KCNN3	*potassium calcium-activated channel subfamily N member 3*	1,758,811	1,780,362	minus	781,978
			PMVK	*phosphomevalonate kinase*	1,781,459	1,784,289	minus	804,626
			PBXIP1	*PBX homeobox interacting protein 1*	1,784,644	1,788,641	minus	807,811
			PYGO2	*pygopus family PHD finger 2*	1,788,639	1,790,498	minus	811,806
			SHC1	*SHC adaptor protein 1*	1,790,787	1,800,935	minus	813,954
			CKS1B	*CDC28 protein kinase regulatory subunit 1B*	1,801,177	1,802,314	plus	824,344
			FLAD1	*flavin adenine dinucleotide synthetase 1*	1,802,652	1,808,111	plus	825,819
			ZBTB7B	*zinc finger and BTB domain containing 7B*	1,812,250	1,830,425	plus	835,417
			DCST2	*DC-STAMP domain containing 2*	1,832,750	1,837,666	minus	855,917
			SMAD4	*SMAD family member 4*	1,838,974	1,844,533	minus	862,141
			CHTOP	*chromatin target of PRMT1*	1,847,078	1,853,476	minus	870,245
			S100A1	*S100 calcium binding protein A1*	1,853,739	1,856,049	minus	876,906
			S100A13	*S100 calcium binding protein A13*	1,857,835	1,859,090	plus	881,002
			S100A14	*S100 calcium binding protein A14*	1,861,081	1,863,210	plus	884,248

			S100A16	*S100 calcium binding protein A16*	1,865,897	1,868,591	plus	889,064
			S100A4	*S100 calcium binding protein A4*	1,869,231	1,871,230	plus	892,398
			S100A6	*S100 calcium binding protein A6*	1,874,323	1,875,575	plus	897,49
			LOC101747386	*protein S100-A9-like*	1,877,071	1,878,016	plus	900,238
			S100A9	*S100 calcium binding protein A9*	1,885,186	1,886,621	plus	908,353
			EDKM	*epidermal differentiation protein containing a KKLIQQ motif*	1,892,914	1,895,414	plus	916,081
			EDQM1	*epidermal differentiation protein containing a glutamine (Q) motif 1*	1,895,773	1,896,542	minus	918,94
			EDQM2	*epidermal differentiation protein containing a glutamine (Q) motif 2*	1,899,100	1,900,320	minus	922,267
			EDWM	*epidermal differentiation protein containing WYDP motif*	1,906,417	1,907,809	minus	929,584
			EDCH5	*epidermal differentiation protein containing cysteine histidine motifs 5*	1,909,483	1,911,230	minus	932,65
			EDMPN1	*epidermal differentiation protein containing a MPN sequence motif 1*	1,912,397	1,913,451	minus	935,564
			EDCRP	*epidermal differentiation cysteine-rich protein*	1,919,906	1,922,026	minus	943,073
			EDCH4	*epidermal differentiation protein containing cysteine histidine motifs 4*	1,931,045	1,932,007	minus	954,212
			EDGH	*epidermal differentiation protein rich in glycine and histidine*	1,940,110	1,942,027	minus	963,277
			LOR1	*loricrin 1*	1,943,612	1,951,026	minus	966,779
			LOR2	*loricrin 2*	1,943,846	1,946,131	minus	967,013
			LOR3	*loricrin 3*	1,952,705	1,955,656	minus	975,872
			EDMTF4	*epidermal differentiation protein starting with MTF motif 4*	1,960,713	1,980,545	plus	983,88
rs317627533	26	4,597,439	*SYT6*	*synaptotagmin 6*	3,808,772	3,837,467	minus	759,972

			TRIM33	*tripartite motif containing 33*	3,844,903	3,869,654	minus	727,785
			BCAS2	*breast carcinoma amplified sequence 2*	3,869,908	3,872,919	minus	724,52
			DENND2C	*DENN domain containing 2C*	3,872,935	3,887,414	minus	710,025
			AMPD1	*adenosine monophosphate deaminase 1*	3,892,276	3,902,376	minus	695,063
			NRAS	*neuroblastoma RAS viral oncogene homolog*	3,906,425	3,912,827	minus	684,612
			CSDE1	*cold shock domain containing E1*	3,912,971	3,930,268	minus	667,171
			SIKE1	*suppressor of IKBKE 1*	3,930,830	3,935,439	minus	662
			BARL	*bile acid receptor-like*	3,941,128	3,952,591	plus	644,848
			SYCP1	*synaptonemal complex protein 1*	3,951,965	3,966,955	plus	630,484
			LOC107049139	*synaptonemal complex protein 1-like*	3,966,969	3,974,901	plus	622,538
			TSHB	*thyroid stimulating hormone beta*	3,974,272	3,987,526	plus	609,913
			TSPAN2	*tetraspanin 2*	3,985,688	4,005,584	minus	591,855
			LOC101747848	*uncharacterized LOC101747848*	4,007,609	4,013,783	plus	583,656
			NGF	*nerve growth factor*	4,027,894	4,050,872	minus	546,567
			LOC101747895	*uncharacterized LOC101747895*	4,059,614	4,065,666	minus	531,773
			LOC101747934	*uncharacterized LOC101747934*	4,066,019	4,077,162	minus	520,277
			FANCE	*Fanconi anemia complementation group E*	4,080,342	4,084,706	minus	512,733
			MKRN3	*makorin ring finger protein 3*	4,084,828	4,086,964	minus	510,475
			PPARD	*peroxisome proliferator activated receptor delta*	4,089,638	4,106,338	minus	491,101
			DEF6	*DEF6, guanine nucleotide exchange factor*	4,108,781	4,120,796	minus	476,643
			ZNF76	*zinc finger protein 76*	4,121,087	4,130,610	minus	466,829
			RPL10A	*ribosomal protein L10a*	4,130,666	4,134,401	plus	463,038

			SCUBE3	*signal peptide, CUB domain and EGF like domain containing 3*	4,135,277	4,165,701	minus	431,738
			TCP11	*t-complex 11*	4,177,656	4,188,605	plus	408,834
			ANKS1A	*ankyrin repeat and sterile alpha motif domain containing 1A*	4,186,369	4,271,993	minus	325,446
			LOC107055188	*uncharacterized LOC107055188*	4,243,866	4,248,941	plus	348,498
			TAF11	*TATA-box binding protein associated factor 11*	4,272,574	4,276,039	plus	321,4
			UHRF1BP1	*UHRF1 binding protein 1*	4,276,310	4,303,901	minus	293,538
			SNRPC	*small nuclear ribonucleoprotein polypeptide C*	4,306,380	4,310,436	minus	287,003
			C26H6orf106	*chromosome 26 C6orf106 homolog*	4,312,154	4,344,017	plus	253,422
			SPDEF	*SAM pointed domain containing ETS transcription factor*	4,353,136	4,359,030	plus	238,409
			PACSIN1	*protein kinase C and casein kinase substrate in neurons 1*	4,359,838	4,375,042	minus	222,397
			RPS10	*ribosomal protein S10*	4,376,915	4,382,439	plus	215
			NUDT3	*nudix hydrolase 3*	4,384,291	4,411,296	plus	186,143
			LOC100858737	*uncharacterized LOC100858737*	4,412,794	4,414,947	plus	182,492
			HMGA1	*high mobility group AT-hook 1*	4,415,782	4,421,656	minus	175,783
			LOC107055185	*uncharacterized LOC107055185*	4,421,016	4,430,830	plus	166,609
			GRM4	*glutamate receptor, metabotropic 4*	4,434,279	4,475,476	plus	121,963
			LOC101750261	*uncharacterized LOC101750261*	4,539,410	4,571,201	plus	26,238
			OPN1MSW	*opsin, green sensitive (rhodopsin-like)*	4,557,195	4,562,805	minus	34,634
			MLN	*motilin*	4,573,764	4,580,079	plus	17,36
			LEMD2	***LEM domain containing 2***	4,584,641	4,597,668	plus	0
			LOC107055184	***uncharacterized LOC107055184***	4,597,433	4,601,335	minus	0
			IP6K3	*inositol hexakisphosphate kinase 3*	4,601,290	4,614,097	plus	3,851

			C26H6ORF125	*chromosome 26 open reading frame, human C6orf125*	4,615,609	4,620,391	plus	18,17
			ITPR3	*inositol 1,4,5-trisphosphate receptor type 3*	4,619,903	4,659,888	minus	22,464
			LOC768477	*uncharacterized LOC768477*	4,669,560	4,675,843	minus	72,121
			BAK1	*BCL2 antagonist/killer 1*	4,677,808	4,689,669	plus	80,369
			LOC107055182	*uncharacterized LOC107055182*	4,690,003	4,698,367	minus	92,564
			TSPO2	*translocator protein 2*	4,698,990	4,704,107	plus	101,551
			LOC107055181	*uncharacterized LOC107055181*	4,707,001	4,710,843	minus	109,562
			APOBEC2	*apolipoprotein B mRNA editing enzyme catalytic subunit 2*	4,710,803	4,718,654	plus	113,364
			OARD1	*O-acyl-ADP-ribose deacylase 1*	4,719,478	4,722,726	minus	122,039
			LOC107055164	*glycine-rich protein DOT1-like*	4,722,793	4,723,646	plus	125,354
			NFYA	*nuclear transcription factor Y subunit alpha*	4,723,085	4,737,464	plus	125,646
			LOC100858470	*uncharacterized LOC100858470*	4,737,878	4,774,348	plus	140,439
			TREM-B1	*triggering receptor expressed on myeloid cells B1*	4,741,376	4,747,862	minus	143,937
			TREM2	*triggering receptor expressed on myeloid cells 2*	4,749,175	4,753,562	minus	151,736
			TREM-B2	*triggering receptor expressed on myeloid cells B2*	4,755,477	4,761,898	minus	158,038
			LOC107055180	*uncharacterized LOC107055180*	4,782,696	4,797,766	plus	185,257
			LOC107055165	*uncharacterized LOC107055165*	4,802,076	4,806,903	plus	204,637
			FOXP4L	*forkhead box protein P4-like*	4,844,332	4,887,023	plus	246,893
			MDFI	*MyoD family inhibitor*	4,891,121	4,906,184	plus	293,682
			TFEB	*transcription factor EB*	4,925,985	4,940,293	minus	328,546
			GASTL	*gastricsin-like*	4,942,094	4,945,050	minus	344,655

			PGC	*progastricsin (pepsinogen C)*	4,946,918	4,950,762	minus	349,479
			FRS3	*fibroblast growth factor receptor substrate 3*	4,952,522	4,965,746	minus	355,083
			PRICKLE4	*prickle planar cell polarity protein 4*	4,966,313	4,973,328	plus	368,874
			LOC101749017	*platelet binding protein GspB-like*	4,973,531	4,985,366	minus	376,092
			TOMM6	*translocase of outer mitochondrial membrane 6*	4,985,455	4,986,384	plus	388,016
			USP49	*ubiquitin specific peptidase 49*	4,986,408	5,019,177	minus	388,969
			LOC107055176	*uncharacterized LOC107055176*	5,019,458	5,022,472	plus	422,019
			MED20	*mediator complex subunit 20*	5,022,173	5,026,778	minus	424,734
			BYSL	*bystin like*	5,026,764	5,031,080	plus	429,325
			CCND3	*cyclin D3*	5,030,661	5,069,048	minus	433,222
			TAF8	*TATA-box binding protein associated factor 8*	5,069,068	5,076,732	plus	471,629
			PIFO	*primary cilia formation*	5,078,237	5,081,253	minus	480,798
			CHIA-M31	*chitinase-M31, acidic*	5,081,386	5,086,043	minus	483,947
			CHIA	*chitinase, acidic*	5,088,647	5,092,767	minus	491,208
			LOC768786	*acidic mammalian chitinase-like*	5,095,653	5,100,518	minus	498,214
			LOC107055174	*uncharacterized LOC107055174*	5,107,408	5,111,368	plus	509,969
			LOC107055171	*uncharacterized LOC107055171*	5,120,914	5,123,009	minus	523,475
			BTG2	*BTG anti-proliferation factor 2*	5,123,154	5,127,216	plus	525,715
			LOC107055173	*uncharacterized LOC107055173*	5,128,663	5,129,912	minus	531,224
			LOC107055172	*uncharacterized LOC107055172*	5,129,960	5,131,921	plus	532,521
			FMOD	*fibromodulin*	5,133,650	5,140,211	minus	536,211
			LOC107055169	*uncharacterized LOC107055169*	5,153,629	5,170,330	minus	556,19
			PRELP	*proline and arginine rich end leucine rich repeat protein*	5,163,841	5,174,857	plus	566,402

			OPTC	*opticin*	5,176,849	5,180,331	plus	579,41
			ATP2B4	*ATPase plasma membrane Ca2+ transporting 4*	5,210,403	5,247,582	plus	612,964
			LOC107055168	*uncharacterized LOC107055168*	5,245,482	5,259,467	minus	648,043
			MIR7454	*microRNA 7454*	5,270,406	5,270,459	minus	672,967
rs314452928	27	104,022	*LOC107055210*	*uncharacterized LOC107055210*	506	1,277	plus	102,745
			LOC107049042	*olfactory receptor 4M1-like*	7,537	8,651	minus	95,371
			LOC768958	*olfactory receptor 6B1-like*	19,365	20,677	minus	83,345
			MROH8	*maestro heat like repeat family member 8*	28,315	37,726	minus	66,296
			LOC107055211	*uncharacterized LOC107055211*	47,117	47,583	minus	56,439
			LOC101751094	*uncharacterized LOC101751094*	58,462	62,958	minus	41,064
			LOC107049117	*uncharacterized LOC107049117*	66,251	68,943	minus	35,079
			LOH11CR2A	*loss of heterozygosity, 11, chromosomal region 2, gene A*	160,44	171,21	plus	56,418
			LOC107055212	*uncharacterized LOC107055212*	160,85	166,792	minus	56,828
			DAD1	*defender against cell death 1*	172,289	175,051	plus	68,267
			IGHVL	*Ig heavy chain Mem5-like*	181,398	731,094	minus	77,376
			LOC101750797	*immunoglobulin omega chain-like*	224,224	584,73	minus	120,202
rs315329074	27	4,528,275	*TRNAI-UAU*	*transfer RNA isoleucine (anticodon UAU)*	4,520,423	4,520,513	plus	7,762
			TRNAQ-UUG	*transfer RNA glutamine (anticodon UUG)*	3,640,823	3,640,894	minus	887,381
			MEOX1	*mesenchyme homeobox 1*	3,530,597	3,536,176	plus	992,099
			ETV4	*ETS variant 4*	3,548,537	3,564,258	plus	964,017
			DHX8	*DEAH-box helicase 8*	3,567,743	3,579,115	minus	949,16
			PHB	*prohibitin*	3,580,883	3,585,030	plus	943,245

			LOC101750197	*uncharacterized LOC101750197*	3,582,478	3,592,058	minus	936,217
			ZNF652	*zinc finger protein 652*	3,592,238	3,620,525	plus	907,75
			PHOSPHO1	*phosphoethanolamine/phosphocholine phosphatase*	3,623,753	3,631,815	plus	896,46
			ABI3	*ABI family member 3*	3,631,245	3,637,401	minus	890,874
			GNGT2	*G protein subunit gamma transducin 2*	3,637,559	3,639,810	plus	888,465
			IGF2BP1	*insulin like growth factor 2 mRNA binding protein 1*	3,648,588	3,672,763	minus	855,512
			GIP	*gastric inhibitory polypeptide*	3,682,114	3,689,763	plus	838,512
			SNF8	*SNF8, ESCRT-II complex subunit*	3,690,040	3,693,337	plus	834,938
			UBE2Z	*ubiquitin conjugating enzyme E2 Z*	3,693,620	3,705,485	minus	822,79
			ATP5G1	*ATP synthase, H+ transporting, mitochondrial Fo complex subunit C1 (subunit 9)*	3,707,200	3,709,533	minus	818,742
			CALCOCO2	*calcium binding and coiled-coil domain 2*	3,709,693	3,719,291	plus	808,984
			HOXB13	*homeobox B13*	3,742,876	3,745,452	plus	782,823
			LOC107055286	*uncharacterized LOC107055286*	3,764,713	3,778,499	plus	749,776
			MIR196A1	*microRNA 196a-1*	3,775,036	3,775,130	plus	753,145
			HOXB9	*homeobox B9*	3,781,877	3,788,281	plus	739,994
			HOXB8	*homeobox B8*	3,795,352	3,796,999	plus	731,276
			HOXB7	*homeobox B7*	3,798,149	3,803,481	plus	724,794
			LOC107055284	*uncharacterized LOC107055284*	3,810,034	3,821,988	minus	706,287
			HOXB6	*homeobox B6*	3,812,716	3,815,203	plus	713,072
			HOXB5	*homeobox B5*	3,818,138	3,820,563	plus	707,712
			MIR10A	*microRNA 10a*	3,834,170	3,834,243	plus	694,032

			HOXB4	*homeobox B4*	3,835,207	3,840,489	plus	687,786
			LOC107055283	*uncharacterized LOC107055283*	3,837,488	3,843,542	minus	684,733
			HOXB3	*homeobox B3*	3,840,562	3,864,857	plus	663,418
			LOC107055285	*uncharacterized LOC107055285*	3,852,258	3,856,707	minus	671,568
			HOXB2	*homeobox B2*	3,867,101	3,871,833	plus	656,442
			LOC107055282	*uncharacterized LOC107055282*	3,868,775	3,870,699	minus	657,576
			HOXB1	*homeobox B1*	3,879,443	3,882,191	plus	646,084
			LOC419994	*src kinase-associated phosphoprotein 1-like*	3,927,113	3,969,088	plus	559,187
			LOC101751838	*uncharacterized LOC101751838*	3,980,545	3,984,567	minus	543,708
			TBKBP1	*TBK1 binding protein 1*	4,013,103	4,024,426	minus	503,849
			KPNB1	*karyopherin subunit beta 1*	4,029,274	4,049,551	minus	478,724
			NPEPPS	*aminopeptidase puromycin sensitive*	4,052,247	4,080,818	minus	447,457
			MRPL45	*mitochondrial ribosomal protein L45*	4,083,516	4,087,147	plus	441,128
			GPR179	*G protein-coupled receptor 179*	4,089,962	4,097,067	minus	431,208
			SOCS7	*suppressor of cytokine signaling 7*	4,098,027	4,106,014	plus	422,261
			SKAP1	*src kinase associated phosphoprotein 1*	4,120,634	4,160,530	plus	367,745
			SNX11	*sorting nexin 11*	4,162,424	4,168,386	minus	359,889
			CBX1	*chromobox 1*	4,168,538	4,176,552	plus	351,723
			NFE2L1	*nuclear factor, erythroid 2 like 1*	4,179,406	4,188,312	minus	339,963
			LOC107055292	*uncharacterized LOC107055292*	4,188,341	4,189,844	plus	338,431
			CDK5RAP3	*CDK5 regulatory subunit associated protein 3*	4,192,066	4,195,013	minus	333,262
			PRR15L	*proline rich 15 like*	4,195,737	4,196,844	plus	331,431
			PNPO	*pyridoxamine 5'-phosphate oxidase*	4,199,228	4,201,514	minus	326,761
			SP2	*Sp2 transcription factor*	4,202,904	4,212,077	minus	316,198

			SP6	*Sp6 transcription factor*	4,219,693	4,222,841	plus	305,434
			SCRN2	*secernin 2*	4,223,661	4,226,254	plus	302,021
			LRRC46	*leucine rich repeat containing 46*	4,226,229	4,229,327	minus	298,948
			MRPL10	*mitochondrial ribosomal protein L10*	4,229,306	4,231,308	plus	296,967
			OSBPL7	*oxysterol binding protein like 7*	4,231,743	4,238,314	plus	289,961
			TBX21	*T-box 21*	4,238,676	4,245,894	minus	282,381
			ARHGAP23	*Rho GTPase activating protein 23*	4,260,935	4,274,769	plus	253,506
			SRCIN1	*SRC kinase signaling inhibitor 1*	4,278,768	4,318,568	minus	209,707
			LOC107055293	*SKI/DACH domain-containing protein 1-like*	4,356,552	4,360,423	minus	167,852
			MIR6663	*microRNA 6663*	4,371,072	4,371,181	minus	157,094
			MLLT6	*MLLT6, PHD finger domain containing*	4,374,478	4,403,841	plus	124,434
			MIR1735	*microRNA 1735*	4,391,229	4,391,307	plus	136,968
			LOC107055294	*polycomb group RING finger protein 2-like*	4,405,220	4,407,907	minus	120,368
			LOC107055296	*protein AF-17-like*	4,410,156	4,419,961	plus	108,314
			CISD3	*CDGSH iron sulfur domain 3*	4,421,720	4,422,397	plus	105,878
			PCGF2	*polycomb group ring finger 2*	4,422,951	4,428,429	minus	99,846
			LOC107055298	*POU domain, class 3, transcription factor 3-like*	4,425,831	4,427,935	plus	100,34
			PSMB3	*proteasome subunit beta 3*	4,430,285	4,433,174	plus	95,101
			PIP4K2B	*phosphatidylinositol-5-phosphate 4-kinase type 2 beta*	4,434,946	4,450,427	minus	77,848
			CWC25	*CWC25 spliceosome associated protein homolog*	4,451,015	4,459,377	minus	68,898
			RPL23	*ribosomal protein L23*	4,463,064	4,465,066	minus	63,209
			LASP1	*LIM and SH3 protein 1*	4,466,693	4,486,609	plus	41,666

			FBXO47	*F-box protein 47*	4,490,226	4,500,566	minus	27,709
			LOC101749109	*uncharacterized LOC101749109*	4,498,009	4,506,617	plus	21,658
			PLXDC1	*plexin domain containing 1*	4,505,312	4,518,001	minus	10,274
			LOC100858629	*dickkopf-related protein 1-like*	4,519,315	4,521,946	plus	6,329
			CACNB1	***calcium voltage-gated channel auxiliary subunit beta 1***	4,521,859	4,534,179	minus	0
			RPL19	*ribosomal protein L19*	4,534,596	4,536,944	plus	6,321
			FBXL20	*F-box and leucine rich repeat protein 20*	4,537,208	4,558,569	minus	8,933
			MED1	*mediator complex subunit 1*	4,558,906	4,572,538	minus	30,631
			CDK12	*cyclin dependent kinase 12*	4,572,918	4,595,934	plus	44,643
			NEUROD2	*neuronal differentiation 2*	4,610,541	4,613,182	minus	82,266
			PPP1R1B	*protein phosphatase 1 regulatory inhibitor subunit 1B*	4,621,254	4,626,595	plus	92,979
			STARD3	*StAR related lipid transfer domain containing 3*	4,626,814	4,643,949	plus	98,539
			TCAP	*titin-cap*	4,644,741	4,646,231	plus	116,466
			PNMT	*phenylethanolamine N-methyltransferase*	4,647,235	4,649,104	plus	118,96
			PGAP3	*post-GPI attachment to proteins 3*	4,649,381	4,652,406	minus	121,106
			ERBB2	*erb-b2 receptor tyrosine kinase 2*	4,653,394	4,662,322	plus	125,119
			MIR6547	*microRNA 6547*	4,660,683	4,660,802	plus	132,408
			MIEN1	*migration and invasion enhancer 1*	4,663,033	4,664,878	minus	134,758
			GRB7	*growth factor receptor bound protein 7*	4,665,681	4,671,472	plus	137,406
			LOC100858293	*retinol dehydrogenase 8-like*	4,671,617	4,674,052	plus	143,342
			IKZF3	*IKAROS family zinc finger 3*	4,676,390	4,700,355	minus	148,115
			ZPBP2	*zona pellucida binding protein 2*	4,700,373	4,706,427	plus	172,098

			LRRC3C	*leucine rich repeat containing 3C*	4,710,386	4,714,334	minus	182,111
			LOC107055297	*basic proline-rich protein-like*	4,713,858	4,715,843	plus	185,583
			ORMDL3	*ORMDL sphingolipid biosynthesis regulator 3*	4,717,225	4,725,376	plus	188,95
			GSDMA	*gasdermin A*	4,725,470	4,730,952	plus	197,195
			PSMD3	*proteasome 26S subunit, non-ATPase 3*	4,731,389	4,735,157	plus	203,114
			CSF3	*colony stimulating factor 3*	4,737,417	4,739,289	plus	209,142
			MIR6884	*microRNA 6884*	4,741,104	4,757,219	minus	212,829
			THRA	*thyroid hormone receptor, alpha*	4,764,287	4,775,504	plus	236,012
			NR1D1	*nuclear receptor subfamily 1 group D member 1*	4,776,457	4,783,436	minus	248,182
			MSL1	*male specific lethal 1 homolog*	4,787,718	4,792,939	plus	259,443
			CASC3	*cancer susceptibility 3*	4,793,935	4,803,203	plus	265,66
			RAPGEFL1	*Rap guanine nucleotide exchange factor like 1*	4,804,082	4,809,927	plus	275,807
			WIPF2	*WAS/WASL interacting protein family member 2*	4,810,433	4,822,471	plus	282,158
			CDC6	*cell division cycle 6*	4,822,688	4,827,078	plus	294,413
			RARA	*retinoic acid receptor alpha*	4,833,202	4,836,052	plus	304,927
			GJD3	*gap junction protein delta 3*	4,836,854	4,839,029	minus	308,579
			TOP2A	*topoisomerase (DNA) II alpha*	4,839,029	4,856,730	minus	310,754
			LOC101747522	*collagen alpha-1(XVIII) chain-like*	4,857,355	4,863,016	minus	329,08
			IGFBP4	*insulin like growth factor binding protein 4*	4,865,485	4,870,779	plus	337,21
			TNS4	*tensin 4*	4,870,793	4,882,163	minus	342,518
			LOC107055315	*uncharacterized LOC107055315*	4,882,026	4,907,719	plus	353,751
			CCR7	*C-C motif chemokine receptor 7*	4,891,171	4,899,901	minus	362,896

			SMARCE1	*SWI/SNF related, matrix associated, actin dependent regulator of chromatin, subfamily e, member 1*	4,907,121	4,921,663	minus	378,846
			KRT222	*keratin 222*	4,920,500	4,929,780	minus	392,225
			LOC107055316	*uncharacterized LOC107055316*	4,922,980	4,937,276	plus	394,705
			KRT12	*keratin 12*	4,932,629	4,939,390	minus	404,354
			KRT20	*keratin 20*	4,939,479	4,943,592	minus	411,204
			KRT23	*keratin 23*	4,953,002	4,962,692	minus	424,727
			KRT15	*keratin 15*	4,965,385	4,969,810	minus	437,11
			KRT19	*keratin 19*	4,972,722	4,977,384	minus	444,447
			LOC420043	*keratin 16-like*	4,983,598	4,987,746	minus	455,323
			LOC100857659	*keratin, type I cytoskeletal 42-like*	4,992,053	4,995,428	minus	463,778
			KRT10	*keratin, type I cytoskeletal 10-like*	4,997,475	5,002,485	minus	469,2
			KRT9L	*keratin, type I cytoskeletal 9-like*	5,008,298	5,012,957	minus	480,023
			LOC772080	*keratin, type I cytoskeletal 17-like*	5,016,347	5,021,212	minus	488,072
			KRTC42L	*keratin, type I cytoskeletal 42-like*	5,025,520	5,030,185	minus	497,245
			LOC771995	*keratin, type I cytoskeletal 42-like*	5,035,336	5,038,163	minus	507,061
			LOC107055312	*uncharacterized LOC107055312*	5,040,540	5,042,410	plus	512,265
			KRT14	*keratin 14*	5,042,180	5,046,007	minus	513,905
			KRT17	*keratin 17*	5,048,947	5,052,819	minus	520,672
			LOC107055313	*uncharacterized LOC107055313*	5,051,305	5,052,029	plus	523,03
			EIF1	*eukaryotic translation initiation factor 1*	5,064,906	5,066,718	plus	536,631
			LOC396365	*preprogastrin*	5,069,166	5,069,814	plus	540,891
			HAP1	*huntingtin associated protein 1*	5,069,870	5,078,795	minus	541,595
			JUP	*junction plakoglobin*	5,081,253	5,096,734	minus	552,978

			P3H4	*prolyl 3-hydroxylase family member 4 (non-enzymatic)*	5,097,792	5,102,079	minus	569,517
			FKBP10	*FK506 binding protein 10*	5,102,602	5,108,343	plus	574,327
			NT5C3B	*5'-nucleotidase, cytosolic IIIB*	5,108,609	5,113,795	minus	580,334
			KLHL10	*kelch like family member 10*	5,113,816	5,117,579	plus	585,541
			KLHL11	*kelch like family member 11*	5,117,750	5,123,733	minus	589,475
			ACLY	*ATP citrate lyase*	5,124,218	5,152,667	minus	595,943
			TTC25	*tetratricopeptide repeat domain 25*	5,149,242	5,156,676	plus	620,967
			CNP	*2',3'-cyclic nucleotide 3' phosphodiesterase*	5,156,868	5,161,795	plus	628,593
			DNAJC7	*DnaJ heat shock protein family (Hsp40) member C7*	5,161,978	5,181,291	minus	633,703
			LOC107055311	*uncharacterized LOC107055311*	5,172,889	5,180,535	plus	644,614
			NKIRAS2	*NFKB inhibitor interacting Ras like 2*	5,181,433	5,183,584	plus	653,158
			ZNF385C	*zinc finger protein 385C*	5,183,603	5,237,098	minus	655,328
			ZNF862L	*zinc finger protein 862-like*	5,198,667	5,206,571	minus	670,392
			LOC107055310	*uncharacterized LOC107055310*	5,223,122	5,224,406	minus	694,847
			DHX58	*DExH-box helicase 58*	5,242,809	5,249,758	minus	714,534
			KAT2A	*lysine acetyltransferase 2A*	5,249,877	5,256,053	minus	721,602
			LOC772158	*heat shock protein 30C-like*	5,256,460	5,257,349	minus	728,185
			HSPB9	*heat shock protein family B (small) member 9*	5,258,006	5,258,998	plus	729,731
			RAB5C	*RAB5C, member RAS oncogene family*	5,260,322	5,270,725	minus	732,047
			KCNH4	*potassium voltage-gated channel subfamily H member 4*	5,271,367	5,280,067	minus	743,092
			HCRT	*hypocretin neuropeptide precursor*	5,280,135	5,281,806	minus	751,86

				PIBPPDD4L	*1-phosphatidylinositol-4,5-bisphosphate phosphodiesterase delta-4-like*	5,281,905	5,292,750	minus	753,63
				GHDC	*GH3 domain containing*	5,294,204	5,298,441	minus	765,929
				STAT5B	*signal transducer and activator of transcription 5B*	5,297,863	5,311,670	minus	769,588
				STAT3	*signal transducer and activator of transcription 3*	5,319,501	5,334,933	minus	791,226
				PTRF	*polymerase I and transcript release factor*	5,335,537	5,350,085	minus	807,262
				ATP6V0A1	*ATPase H+ transporting V0 subunit a1*	5,352,689	5,381,019	plus	824,414
				NAGLU	*N-acetylglucosaminidase, alpha*	5,381,264	5,384,665	plus	852,989
				HSD17B1	*hydroxysteroid 17-beta dehydrogenase 1*	5,384,880	5,386,410	plus	856,605
				MLX	*MLX, MAX dimerization protein*	5,390,278	5,394,161	plus	862,003
				PSMC3IP	*PSMC3 interacting protein*	5,393,341	5,397,274	minus	865,066
				FAM134C	*family with sequence similarity 134 member C*	5,397,367	5,405,515	minus	869,092
				TUBG1	*tubulin gamma 1*	5,405,589	5,413,030	plus	877,314
				LOC107055309	*uncharacterized LOC107055309*	5,407,102	5,408,044	minus	878,827
				PLEKHH3	*pleckstrin homology, MyTH4 and FERM domain containing H3*	5,413,790	5,421,146	minus	885,515
				CCR10	*C-C motif chemokine receptor 10*	5,422,621	5,427,142	minus	894,346
				CNTNAP1	*contactin associated protein 1*	5,427,307	5,435,737	plus	899,032
				EZH1	*enhancer of zeste 1 polycomb repressive complex 2 subunit*	5,435,970	5,451,101	minus	907,695
				RAMP2	*receptor activity modifying protein 2*	5,453,137	5,454,994	plus	924,862
				VPS25	*vacuolar protein sorting 25 homolog*	5,455,180	5,457,660	plus	926,905

			WNK4	*WNK lysine deficient protein kinase 4*	5,461,875	5,473,473	plus	933,6
			COA3	*cytochrome c oxidase assembly factor 3*	5,473,589	5,474,238	minus	945,314
			CNTD1	*cyclin N-terminal domain containing 1*	5,474,297	5,478,907	plus	946,022
			BECN1	*beclin 1*	5,478,573	5,483,381	minus	950,298
			PSME3	*proteasome activator subunit 3*	5,483,531	5,490,236	plus	955,256
			AOC3	*amine oxidase, copper containing 3*	5,490,347	5,504,360	plus	962,072
			G6PC	*glucose-6-phosphatase catalytic subunit*	5,506,845	5,510,258	plus	978,57
			AARSD1	*alanyl-tRNA synthetase domain containing 1*	5,511,314	5,515,677	minus	983,039
			PTGES3L	*prostaglandin E synthase 3 (cytosolic)-like*	5,515,779	5,517,799	minus	987,504
			RUNDC1	*RUN domain containing 1*	5,518,274	5,520,926	plus	989,999
			RPL27	*ribosomal protein L27*	5,521,996	5,524,344	plus	993,721
			IFI35	*interferon-induced protein 35*	5,524,907	5,535,384	plus	996,632
* Note: Positions are based on Gallus gallus 5.0 genome assembly. Genes including the significant marker are shown in bold.								
