# Supplementary material for: Discovery and characterization of functional modules associated with body weight in broilers
Source: Sci Rep. 2019 Jun 24;9:9125. doi: 10.1038/s41598-019-45520-5 (PMC6591351; doi:10.1038/s41598-019-45520-5)
Supplement: Supplementary file 2 — Table S2 [file 41598_2019_45520_MOESM2_ESM.pdf]

Discovery and characterization of functional modules associated with body weight in broilers

Eirini Tarsani[1*], Andreas Kranis[2,3], Gerasimos Maniatis[2], Santiago Avendano[2], Ariadne L. Hager-Theodorides[1], Antonios Kominakis[1]

[1]Department of Animal Science and Aquaculture, Agricultural University of Athens, Iera Odos 75, 11855, Athens, Greece

[2]Aviagen Ltd., Newbridge, Midlothian EH28 8SZ, UK

[3] The Roslin Institute, University of Edinburgh, EH25 9RG, Midlothian, United Kingdom

*corresponding author: etarsani@aua.gr

Table S2: Published QTL/associations related to growth traits in the searched genomic regions. In bold are shown the QTL that included all candidate genes in the predefined distances.

SNP ID	GGA	Distance (bp)	Number of QTL	QTL (bp)*	QTL type	QTL IDs*
rs13923872	1	613,054	20	**37,278,942 - 128,288,555**	**Chest width**	**16706**
				**18,054,807 - 171,631,116**	**Visceral fat weight**	**17319**
				**18,054,807 - 171,717,298**	**Total white fat weight**	**17332**
				**25,724,479 - 171,631,116**	**Subcutaneous neck fat weight**	**17325**

				**18,054,807 - 196,202,543**	**Body weight**	**1797**
				**37,278,942 - 133,528,161**	**Body weight (140 days)**	**55919**
				**18,054,807 - 171,631,116**	**Body weight (140 days)**	**17076**
				**18,054,807 - 171,631,116**	**Carcass weight**	**17110**
				**100,051,042 - 123,004,362**	**Breast muscle weight**	**9410**
				**2,420,814 - 171,631,116**	**Shank length**	**9409**
				**106,349,346 - 123,007,835**	**Carcass fat content**	**17119**
				113,159,926 - 128,288,555	Shank weight	14341
				113,159,926 - 128,288,555	Femur weight	14342
				**18,054,807 - 172,427,968**	**Spleen weight**	**1851**
				**18,054,807 - 171,631,116**	**Growth (70-105 days)**	**55937**
				**18,054,807 - 168,151,247**	**Abdominal fat weight**	**6858**
				**18,054,807 - 171,631,116**	**Shank length**	**9294**
				113,159,926 - 115,848,566	Breast muscle weight	13385
				**111,368,640 - 164,599,096**	**Body weight (35 days)**	**14355**

				**6,580,919 - 171,631,116**	**Subcutaneous fat thickness**	**14359**
rs312691174	4	650,472	14	**17,148,380 - 81,264,760**	**Body weight (168 days)**	**24875**
				**17,148,380 - 81,264,760**	**Body weight (21 days)**	**24842**
				**17,148,380 - 81,264,760**	**Body weight (336 days)**	**24883**
				**17,148,380 - 81,264,760**	**Body weight (42 days)**	**24855**
				**17,148,380 - 81,264,760**	**Average daily gain**	**24899, 24905,24911**
				**17,148,380 - 81,264,760**	**Body weight (84 days)**	**24866**
				**17,148,380 - 81,264,760**	**Body weight (504 days)**	**24890**
				**4,964,691 - 87,025,255**	**Visceral fat weight**	**17321**
				**10,768,639 - 91,268,419**	**Average daily gain**	**24914**
				**18,357,474 - 31,942,137**	**Shank length**	**9295**
				**18,354,191 - 37,883,325**	**Thigh muscle weight**	**9395**
				**18,354,191 - 47,647,218**	**Drumstick and thigh muscle weight**	**13404**
				**17,148,380 - 81,264,760**	**Body weight (day of first egg)**	**14457, 14464, 14470**
				**17,148,380 - 81,264,760**	**Head percentage**	**15571**
rs15608447	4	718,407	36	67,546,750 - 67,546,790	Body weight (28 days)	65710

				**17,148,380 - 81,264,760**	**Body weight (168 days)**	**24875**
				**47,647,218 - 89,464,128**	**Body weight**	**200.820.152.016**
				**17,148,380 - 81,264,760**	**Body weight (21 days)**	**24842**
				**17,148,380 - 81,264,760**	**Body weight (336 days)**	**24883**
				**47,647,218 - 89,464,128**	**Carcass weight**	**2012**
				**17,148,380 - 81,264,760**	**Body weight (42 days)**	**24855**
				**17,148,380 - 81,264,760**	**Average daily gain**	**24899, 24905, 24911**
				**17,148,380 - 81,264,760**	**Body weight (84 days)**	**24866**
				**47,647,218 - 89,464,128**	**Liver weight**	**2017**
				**52,604,411 - 82,619,142**	**Growth (14-28 days)**	**12499**
				**17,148,380 - 81,264,760**	**Body weight (504 days)**	**24890**
				**48,804,413 - 85,154,534**	**Growth (28-42 days)**	**12500**
				**52,191,247 - 89,464,128**	**Abdominal fat percentage**	**9421**
				**48,404,949 - 82,619,142**	**Growth (0-14 days)**	**12498**
				**52,535,768 - 70,787,114**	**Shank length**	**9286**
				**49,665,708 - 85,877,678**	**Total white fat weight**	**17334**

				30,906,204 - 83,247,658	Tibia width	2035
				61,970,484 - 83,247,658	Tibia width	2038
				62,452,715 - 89,022,456	Growth (42-56 days)	12501
				4,964,691 - 87,025,255	Visceral fat weight	17321
				62,331,035 - 82,550,230	Pectoralis major weight	2041
				31,561,525 - 89,318,267	Body weight (35 days)	55905
				32,974,594 - 89,318,267	Growth (0-35 days)	55930
				47,647,218 - 89,464,128	Drumstick muscle weight	2057
				10,768,639 - 91,268,419	Average daily gain	24914
				47,647,218 - 89,464,128	Drumstick weight	2059
				47,647,218 - 89,464,128	Wing weight	2060
				47,647,218 - 89,464,128	Body weight (42 days)	9759
				47,647,218 - 89,464,128	Body weight (63 days)	9760
				47,647,218 - 89,464,128	Growth (21-42 days)	9761
				47,647,218 - 89,464,128	Growth (42-63 days)	9762
				62,331,035 - 82,299,229	Shank length	11795

				**47,647,218 - 89,464,128**	**Skin fat weight**	**12636**
				**47,647,218 - 89,464,128**	**Drumstick and thigh muscle weight**	**13395**
				**17,148,380 - 81,264,760**	**Body weight (day of first egg)**	**14457, 14464, 14470**
rs318199727	10	737,906	11	13,330,009 - 13,330,034	Dressing percentage	57550
				13,330,009 - 13,330,034	Breast muscle percentage	57551
				13,329,989 - 13,330,029	Breast muscle percentage	57547
				13,329,989 - 13,330,029	Drumstick and thigh muscle percentage	57548
				13,329,989 - 13,330,029	Abdominal fat percentage	57549
				**692,555 -20,423,025**	**Carcass weight**	**17113**
				**1,541,735 - 16,171,711**	**Body weight (140 days)**	**55923**
				**2,357,400 - 17,864,188**	**Body weight (35 days)**	**55907**
				**692,555 -20,423,025**	**Body weight (70 days)**	**55911**
				**2,552,841 - 18,059,263**	**Growth (0-35 days)**	**55931**
				**4,410,690 - 18,434,155**	**Body weight (105 days)**	**55917**
rs318098582	11	300,257	9	**1,133,281 - 19,983,730**	**Body weight (140 days)**	**55924**
				**18,193,544 - 20,208,550**	**Thigh meat-to-bone ratio**	**6736**

				**18,193,544 - 20,208,550**	**Body weight (40 days)**	**6737**
				**953,174 -20,208,550**	**Body weight (140 days)**	**17080**
				**6,823,128 - 20,208,550**	**Carcass weight**	**17114**
				**12,510,855 - 20,208,550**	**Carcass weight**	**17088**
				**6,910,612 - 20,208,550**	**Spleen weight**	**2287**
				18,193,544 - 18,870,770	Body weight	2284, 2285
				18,642,683 - 18,686,657	Growth (8-46 days)	9519
rs317945754	15	935,183	21	3,731,712 -3,769,767	Spleen weight	2349
				3,731,712 -3,769,767	Body weight (42 days)	9727
				3,731,712 -3,769,767	Carcass weight	9728
				3,731,712 -3,769,767	Spleen percentage	12588
				2,812,987 - 10,689,472	Body weight (336 days)	24887
				4,236,686 -4,265,310	Abdominal fat weight	11995
				**1,931,502 -7,215,657**	**Visceral fat weight**	**17323**
				**2,519,182 -7,215,657**	**Subcutaneous neck fat weight**	**17331**
				3,749,008 -7,973,093	Drumstick and thigh percentage	15586
				3,749,008 -7,973,093	Abdominal fat weight	2337
				3,749,008 -7,973,093	Abdominal fat percentage	2339, 2340
				3,749,008 -8,228,905	Body weight (35 days)	3355
				**1,931,502 -7,215,657**	**Total white fat weight**	**17337**
				2,812,987 - 10,689,472	Liver weight	2348

				**2,403,639 - 10,689,472**	**Abdominal fat percentage**	**9450**
				**1,931,502 - 10,689,472**	**Abdominal fat weight**	**9451**
				2,812,987 - 10,689,472	Abdominal fat weight	2347, 12631
				3,749,008 - 10,689,472	Body weight (46 days)	6648
				3,749,008 - 10,689,472	Growth (8-46 days)	6649
				2,812,987 - 10,689,472	Fat distribution	12645
				**1,931,502 -9,638,429**	**Breast muscle weight**	**9449**
rs316794400	22	26,589	1	QTL: 3,625,173-4,599,266 bp could not be remapped from Gallus gallus 4 to Gallus gallus 5.0 assembly by NCBI Genome Remapping Service	Breast muscle percentage	95429
rs317627533	26	773,988	6	3,118,976 -4,116,802	Body weight (28 days)	95418
				1,263,919 -4,918,464	Body weight (63 days)	9453
				2,499,704 -4,918,464	Shank weight	2383
				4,610,791 -4,624,276	Liver percentage	2385

				QTL: 4,715,796-108,192,374 bp could not be remapped from Gallus gallus 4 to Gallus gallus 5.0 assembly by NCBI Genome Remapping Service	Abdominal fat weight	30883
				4,873,346 -4,886,832	Breast muscle weight	6957
rs314452928	27	140,067	3	QTL: 81,131-81,301 bp could not be remapped from Gallus gallus 4 to Gallus gallus 5.0 assembly by NCBI Genome Remapping Service	Growth (105-140 days)	55944
				54,597 -4,520,058	Growth (0-35 days)	55932
				54,597 -4,520,058	Body weight (35 days)	55906
rs315329074	27	998,553	65	3,834,510 -3,834,550	Shank length	66068, 66069, 66070
				3,363,708 -3,363,748	Shank length	66067
				3,971,422 -3,971,462	Shank circumference	66063
				3,564,173 -3,564,213	Shank circumference	66065
				3,624,903 -3,624,943	Shank circumference	66064, 66066
				3,869,461 -3,869,501	Shank length	66071
				3,456,748 -3,456,788	Abdominal fat weight	66072
				1,798,380 -3,707,375	Abdominal fat weight	11817, 11809

				1,798,380 -3,707,375	Abdominal fat percentage	11820
				1,798,380 -3,707,375	Carcass fat content	17135, 17126
				1,798,380 -3,707,375	Head percentage	15599
				1,798,380 -3,707,375	Body weight	2406, 2407
				1,798,380 -3,707,375	Body weight (1 day)	7178
				1,798,380 -3,707,375	Body weight (41 days)	7186
				1,365,641 -4,520,058	Humerus length	2397
				3,522,988 -3,534,446	Body weight (112 days)	9521
				3,522,988 -3,534,446	Body weight (200 days)	9522
				3,522,988 -3,534,446	Growth (46-112 days)	9523
				1,365,641 -4,520,058	Body weight	2410
				1,809,980 -3,707,375	Body weight (35 days)	3356
				1,809,980 -3,707,375	Abdominal fat percentage	3354
				1,798,380 -3,707,375	Carcass protein content, dry matter basis	17124
				1,798,380 -3,707,375	Carcass fat content, dry matter basis	17125
				3,701,574 -3,713,173	Body weight (42 days)	9775
				3,701,574 -3,713,173	Growth (21-42 days)	9776
				3,701,574 -3,713,173	Body weight (day of first egg)	14459, 14466, 14473
				3,701,574 -3,713,173	Body weight (168 days)	24878
				3,701,574 -3,713,173	Body weight (336 days)	24888
				3,701,574 -3,713,173	Body weight (504 days)	24892
				3,701,574 -3,713,173	Average daily gain	24907
				3,707,375 -3,968,049	Body weight	2404, 2405

				3,707,375 -4,520,058	Thigh weight	2411
				3,707,375 -4,520,058	Wing weight	2412
				3,788,374 -3,889,766	Drumstick and thigh weight	11920
				3,788,374 -3,889,766	Drumstick and thigh percentage	11921
				3,788,374 -3,889,766	Abdominal fat percentage	11934
				3,788,374 -3,889,766	Pectoralis major percent	11950
				3,204,318 -4,520,058	Shank weight	2413
				1,798,380 -3,707,375	Body weight (112 days)	6652
				1,798,380 -3,707,375	Body weight (200 days)	6653
				1,798,380 -3,707,375	Growth (46-112 days)	6654
				1,798,380 -3,379,175	Femur length	6778
				1,798,380 -3,707,375	Shank weight percentage	15567
				2,263,107 -4,520,058	Carcass weight	17116
				404,762 -4,520,058	Wing weight	17109
				2,780,009 -4,520,058	Body weight (105 days)	55918
				54,597 -4,520,058	Growth (0-35 days)	55932
				1,365,641 -4,520,058	Body weight	2409
				54,597 -4,520,058	Body weight (35 days)	55906
				2,454,458 -4,520,058	Body weight (140 days)	55926
				2,639,460 -4,520,058	Body weight (70 days)	55912
				3,707,375 -4,520,058	Body weight (35 days)	7159
				1,850,810 -4,520,058	Carcass weight	17090
				2,639,460 -4,520,058	Growth (35-70 days)	55936
				4,377,710 -4,389,305	Shank length	9288

				3,707,375 -4,520,058	Shank weight percentage	15595
				2,390,652 -4,520,058	Breast muscle weight	17096
				3,597,175 -4,520,058	Drumstick and thigh weight	17105
				3,707,375 -4,520,058	Intramuscular fat	3360
				2,141,304 -4,520,058	Body weight (140 days)	17084
				3,707,375 -4,520,058	Body weight	2408
				**3,788,374 -5,629,582**	**Thigh percentage**	**30886**
				5,159,872 -5,171,472	Body weight (56 days)	12395
				5,159,872 -5,171,472	Body weight (hatch)	16623
				5,159,872 -5,171,472	Body weight (300 days)	16624
*Note: The positions of QTL were remapped to Gallus_gallus-5.0 genome assembly using NCBI database (https://www.ncbi.nlm.nih.gov/genome/tools/remap)						
