# Supplementary material for: Discovery and characterization of functional modules associated with body weight in broilers
Source: Sci Rep. 2019 Jun 24;9:9125. doi: 10.1038/s41598-019-45520-5 (PMC6591351; doi:10.1038/s41598-019-45520-5)
Supplement: Supplementary file 3 — Table S3 [file 41598_2019_45520_MOESM3_ESM.pdf]

Discovery and characterization of functional modules associated with body weight in broilers

Eirini Tarsani[1*], Andreas Kranis[2,3], Gerasimos Maniatis[2], Santiago Avendano[2], Ariadne L. Hager-Theodorides[1], Antonios Kominakis[1]

[1]Department of Animal Science and Aquaculture, Agricultural University of Athens, Iera Odos 75, 11855, Athens, Greece

[2]Aviagen Ltd., Newbridge, Midlothian EH28 8SZ, UK

[3] The Roslin Institute, University of Edinburgh, EH25 9RG, Midlothian, United Kingdom

*corresponding author: etarsani@aua.gr

Table S3: Genes and chromosomes per module.

**Module ID**	**GGA**	**Gene ID**
module1	4	*NIPAL1*
module2	27	*GPR179*
module2	4	*ZAR1*
module2	4	*PCDH18*
module2	26	*DENND2C*
module2	4	*LRRC66*
module2	25	*NES*
module2	10	*TICRR*
module2	11	*CIDEC*
module2	4	*NOCT*
module2	27	*ARHGAP23*
module2	26	*OPTC*
module2	27	*KRT222*
module2	4	*GABRA4*
module2	26	*IP6K3*
module2	25	*LIM2*
module2	15	*ADGRD1*

module2	15	*TMEM132C*
module2	10	*RHCG*
module2	25	*BCAN*
module2	26	*PIFO*
module2	27	*LRRC46*
module2	4	*GABRG1*
module2	27	*KLHL10*
module2	27	*LRRC3C*
module2	27	*TNS4*
module2	27	*RAPGEFL1*
module2	4	*MGARP*
module2	26	*PACSIN1*
module2	15	*PIWIL1*
module2	15	*TMEM132D*
module2	27	*HOXB8*
module2	26	*TCP11*
module2	26	*FRS3*
module2	27	*HOXB4*
module2	27	*GNGT2*
module2	25	*SLAMF8*
module2	27	*FKBP10*
module2	27	*GSDMA*
module2	4	*CORIN*
module2	26	*CHIA*
module2	25	*TMEM79*
module2	4	*SPATA18*
module2	27	*KCNH4*
module2	27	*HOXB9*
module2	26	*SCUBE3*
module2	25	*RHBG*
module2	4	*RASL11B*
module2	11	*SLC22A31*
module2	26	*SYT6*
module2	25	*INSRR*
module2	25	*S100A14*
module2	4	*CWH43*
module2	4	*SLC10A4*
module2	26	*USP49*
module2	27	*CCR10*
module2	11	*ZFPM1*
module2	25	*HAPLN2*
module2	1	*SYTL5*
module2	25	*S100A16*
module2	11	*IL17C*

module2	15	*TMEM132B*
module2	11	*ZNF469*
module2	27	*TTC25*
module2	27	*ZPBP2*
module2	25	*LRRC71*
module2	25	*PEAR1*
module2	27	*GJD3*
module2	25	*DCST2*
module2	27	*TBKBP1*
module2	27	*HCRT*
module2	1	*SRPX*
module2	27	*G6PC*
module2	10	*SV2B*
module2	27	*THRA*
module2	10	*PLIN1*
module2	25	*SV2A*
module2	26	*TSPO2*
module2	25	*SLAMF1*
module2	26	*GRM4*
module2	26	*PRELP*
module2	27	*MEOX1*
module2	10	*MFGE8*
module2	1	*PRRG1*
module2	26	*SPDEF*
module2	1	*XK*
module2	26	*SYCP1*
module2	26	*MKRN3*
module2	27	*RAMP2*
module2	15	*STX2*
module2	4	*TEC*
module2	27	*HOXB6*
module2	27	*HAP1*
module2	11	*CBFA2T3*
module2	27	*NEUROD2*
module2	15	*FZD10*
module2	4	*GABRB1*
module2	26	*MLN*
module2	27	*HOXB2*
module2	26	*MDFI*
module2	26	*FMOD*
module2	27	*AOC3*
module2	25	*CD48*
module2	27	*P3H4*
module2	27	*GIP*

module2	27	*KRT15*
module2	27	*HSD17B1*
module2	27	*HOXB13*
module2	22	*IL1B*
module2	26	*TSHB*
module2	27	*CNTNAP1*
module2	26	*AMPD1*
module2	27	*KRT17*
module2	26	*NGF*
module2	25	*NHLH1*
module2	25	*CRABP2*
module2	27	*IGFBP4*
module2	4	*SLC7A11*
module2	27	*KRT20*
module2	4	*SGCB*
module2	27	*PNMT*
module2	10	*ACAN*
module2	4	*CNGA1*
module2	25	*RAB25*
module2	26	*PGC*
module2	1	*OTC*
module2	27	*KRT12*
module2	27	*HOXB1*
module2	25	*ZBTB7B*
module2	25	*CHRNB2*
module2	4	*GABRA2*
module2	15	*RIMBP2*
module2	27	*HOXB7*
module2	27	*HOXB5*
module2	10	*RLBP1*
module2	25	*S100A13*
module2	26	*TREM2*
module2	27	*PTRF*
module2	11	*CDH15*
module2	25	*PLEKHO1*
module2	27	*PRR15L*
module2	27	*CACNB1*
module2	27	*PPP1R1B*
module2	27	*KRT23*
module2	25	*OTUD7B*
module2	27	*WNK4*
module2	10	*HAPLN3*
module2	25	*S100A11*
module2	22	*ANXA4*

module2	27	*CCR7*
module2	27	*KLHL11*
module2	25	*ETV3*
module2	11	*CYBA*
module2	1	*CYBB*
module2	25	*S100A9*
module2	27	*SKAP1*
module2	1	*RPGR*
module2	25	*MTMR11*
module2	25	*SH2D2A*
module2	27	*IKZF3*
module2	1	*MED14*
module2	27	*HOXB3*
module2	27	*PLXDC1*
module2	26	*TSPAN2*
module2	27	*ORMDL3*
module2	26	*ATP2B4*
module2	25	*CD244*
module2	15	*SLC15A4*
module2	27	*TBX21*
module2	27	*PGAP3*
module2	25	*S100A6*
module2	27	*OSBPL7*
module2	15	*GLT1D1*
module2	26	*BTG2*
module2	4	*TXK*
module2	1	*TSPAN7*
module2	25	*KCNN3*
module2	27	*KRT14*
module2	27	*KRT19*
module2	27	*CSF3*
module2	25	*S100A1*
module2	25	*S100A4*
module2	15	*MMP17*
module2	25	*LY9*
module2	10	*NTRK3*
module2	25	*CRNN*
module2	27	*NR1D1*
module2	27	*ABI3*
module3	27	*FBXO47*
module3	27	*AARSD1*
module3	27	*PHOSPHO1*
module3	22	*NT5DC4*
module3	10	*KIF7*

module3	4	*NFXL1*
module3	27	*PLEKHH3*
module3	25	*SHE*
module3	27	*SRCIN1*
module3	25	*KIRREL*
module3	27	*PCGF2*
module3	11	*SPG7*
module3	4	*ATP10D*
module3	26	*ANKS1A*
module3	27	*FBXL20*
module3	27	*NT5C3B*
module3	27	*RUNDC1*
module3	1	*BCOR*
module3	27	*MLLT6*
module3	27	*STAT5B*
module3	27	*ERBB2*
module3	27	*SOCS7*
module4	27	*MROH8*
module4	10	*AGBL1*
module4	25	*ETV3L*
module4	4	*RAB33B*
module4	25	*RAB2B*
module4	11	*CTU2*
module4	11	*GALNS*
module4	27	*VWA5A*
module4	1	*DYNLT3*
module4	26	*PPARD*
module4	26	*UHRF1BP1*
module4	1	*ATP6AP2*
module4	4	*FRYL*
module4	26	*SIKE1*
module4	4	*USP46*
module4	26	*NRAS*
module4	4	*ELF2*
module4	27	*ETV4*
module5	11	*PABPN1L*
module5	25	*VANGL2*
module5	27	*PTGES3L*
module5	10	*DET1*
module5	25	*MEX3A*
module5	4	*SCFD2*
module5	26	*TOMM6*
module5	10	*MRPL46*
module5	15	*AACS*

module5	11	*ACSF3*
module5	10	*AEN*
module5	25	*RRNAD1*
module5	26	*FANCE*
module5	25	*PYGO2*
module5	27	*HSPB9*
module5	27	*IGF2BP1*
module5	22	*CKAP2L*
module5	25	*BGLAP*
module5	15	*ULK1*
module5	25	*NTRK1*
module5	27	*PSMC3IP*
module5	27	*TCAP*
module5	26	*BCAS2*
module5	10	*KLHL25*
module5	10	*FANCI*
module5	26	*APOBEC2*
module5	26	*BYSL*
module5	27	*TUBG1*
module5	27	*MRPL45*
module5	26	*PRICKLE4*
module5	27	*GRB7*
module5	26	*ZNF76*
module5	11	*MVD*
module5	26	*NFYA*
module5	26	*TAF11*
module5	27	*RPL23*
module5	27	*STARD3*
module5	27	*RARA*
module5	27	*SP2*
module5	25	*ARHGEF11*
module5	25	*CHTOP*
module5	25	*FLAD1*
module5	25	*MEF2D*
module5	27	*SMARCE1*
module5	27	*WIPF2*
module5	25	*CCT3*
module5	4	*COMMD8*
module5	27	*CASC3*
module5	26	*LEMD2*
module5	25	*MRPL24*
module5	26	*RPS10*
module5	26	*RPL10A*
module5	25	*ILF2*

module5	27	*RPL19*
module5	27	*CBX1*
module5	27	*ATP5G1*
module5	26	*SNRPC*
module5	27	*PSMB3*
module5	25	*ADAR*
module5	25	*PMVK*
module5	27	*EIF1*
module5	25	*VPS45*
module5	26	*CSDE1*
module5	27	*PSMD3*
module5	25	*PRCC*
module5	27	*ATP6V0A1*
module5	25	*COPA*
module5	27	*ACLY*
module5	25	*PBXIP1*
module5	25	*GPATCH4*
module5	25	*NCSTN*
module5	25	*ANP32E*
module5	27	*NFE2L1*
module5	27	*PHB*
module5	26	*NUDT3*
module5	26	*MED20*
module5	27	*MSL1*
module5	27	*BECN1*
module5	27	*NPEPPS*
module5	25	*SMG5*
module5	27	*EZH1*
module5	26	*OARD1*
module5	27	*MED1*
module5	26	*TRIM33*
module5	27	*MRPL10*
module5	27	*UBE2Z*
module5	27	*LASP1*
module5	27	*KRT10*
module5	1	*USP9X*
module5	25	*NAXE*
module5	27	*FAM134C*
module5	15	*EP400*
module5	10	*MRPS11*
module5	25	*UBE2Q1*
module5	27	*PIP4K2B*
module5	26	*TFEB*
module5	25	*INTS3*

module5	10	*POLG*
module5	27	*CNP*
module5	27	*DNAJC7*
module5	27	*MLX*
module5	11	*ANKRD11*
module5	10	*AKAP13*
module5	27	*SNF8*
module5	25	*LAMTOR2*
module5	4	*FIP1L1*
module5	4	*NAA15*
module5	27	*SNX11*
module5	25	*SNAPIN*
module5	27	*NKIRAS2*
module5	1	*MID1IP1*
module5	11	*TRAPPC2L*
module5	27	*COA3*
module5	4	*OCIAD1*
module5	4	*SLAIN2*
module5	11	*BANP*
module5	27	*CISD3*
module5	27	*MIEN1*
module5	26	*TAF8*
module5	11	*ZC3H18*
module5	25	*SHC1*
module5	25	*SMAD4*
module5	25	*CKS1B*
module5	27	*RPL27*
module5	27	*TOP2A*
module5	27	*DAD1*
module5	11	*APRT*
module5	26	*CCND3*
module5	11	*PIEZO1*
module5	26	*HMGA1*
module5	25	*SF3B4*
module5	25	*HDGF*
module5	27	*CALCOCO2*
module5	27	*KAT2A*
module5	15	*RAN*
module5	27	*JUP*
module5	25	*IL6R*
module5	27	*CDC6*
module5	27	*PSME3*
module5	22	*SLC20A1*
module5	27	*KPNB1*

module5	26	*BAK1*
module5	27	*STAT3*
module5	4	*DCUN1D4*
module5	27	*DHX8*
module5	15	*SFSWAP*
module5	27	*VPS25*
module5	25	*UBQLN4*
module5	27	*RAB5C*
module5	11	*CDT1*
module5	15	*PUS1*
module5	27	*CDK12*
module6	27	*ZNF385C*
module6	27	*SP6*
module6	1	*LANCL3*
module6	27	*CNTD1*
module6	27	*PNPO*
module6	27	*DHX58*
module6	10	*PEX11A*
module6	27	*GHDC*
module6	10	*ABHD2*
module6	27	*NAGLU*
module6	26	*ITPR3*
module6	26	*DEF6*
module6	27	*CWC25*
module6	11	*RNF166*
module6	27	*CDK5RAP3*
module6	25	*GLMP*
module6	27	*SCRN2*
module6	4	*SETD7*
module6	27	*IFI35*
module6	4	*MGST2*
module6	4	*MAML3*
module6	27	*ZNF652*
