# Supplementary material for: Discovery and characterization of functional modules associated with body weight in broilers
Source: Sci Rep. 2019 Jun 24;9:9125. doi: 10.1038/s41598-019-45520-5 (PMC6591351; doi:10.1038/s41598-019-45520-5)
Supplement: Supplementary file 4 — Table S4 [file 41598_2019_45520_MOESM4_ESM.pdf]

Discovery and characterization of functional modules associated with body weight in broilers

Eirini Tarsani[1*], Andreas Kranis[2,3], Gerasimos Maniatis[2], Santiago Avendano[2], Ariadne L. Hager-Theodorides[1], Antonios Kominakis[1]

[1]Department of Animal Science and Aquaculture, Agricultural University of Athens, Iera Odos 75, 11855, Athens, Greece

[2]Aviagen Ltd., Newbridge, Midlothian EH28 8SZ, UK

[3] The Roslin Institute, University of Edinburgh, EH25 9RG, Midlothian, United Kingdom

*corresponding author: etarsani@aua.gr

Table S4: Significantly enriched GO biological processes (BPs) per each module. GO BP terms in bold are associated with development. None enriched GO BP was found for module_6.

**Module_ ID**	**GO_ID**	**GO BP term**	**Considered relevant to BW as a subclass or child term of the developmental process or growth parent term**	**P-value**	**Number of genes**	**Associated genes found**
module_2	**GO:0048704**	**embryonic skeletal system morphogenesis**	yes	0.00000000483	9	*HOXB3*, *MDFI*, *HOXB4*, *HOXB1*, *HOXB2*, *HOXB7*, *HOXB8*, *HOXB5*, *HOXB6*
module_2	**GO:0009952**	**anterior/posterior pattern specification**	yes	0.000000125	10	*HOXB3*, *HOXB4*, *HOXB1*, *HOXB2*, *BTG2*, *HOXB7*, *HOXB8*, *HOXB5*, *HOXB6*, *HOXB9*
module_2	**GO:0007417**	**central nervous system development**	yes	0.001	7	*HAPLN2*, *NES*, *HAPLN3*, *GABRA4*, *ACAN*, *BCAN*, *NHLH1*
module_2	**GO:0007275**	**multicellular organism development**	yes	0.003	14	*CSF3*, *ZBTB7B*, *ZAR1*, *TBX21*, *HOXB3*, *FZD10*, *TCP11*, *HOXB1*, *HOXB2*, *HOXB7*, *HOXB8*, *MEOX1*, *PIWIL1*, *SPDEF*
module_2	**GO:0030851**	**granulocyte differentiation**	yes	0.005	3	*CSF3*, *ZFPM1*, *CBFA2T3*
module_2	GO:0042340	keratan sulfate catabolic process	no	0.006	3	*FMOD*, *ACAN*, *PRELP*

module_2	GO:0006811	ion transport	no	0.099	6	*CYBB, GABRA2, WNK4, GABRB1, CHRNB2, SLC15A4*
module_2	**GO:0008544**	**epidermis development**	yes	0.011	5	*KRT17, KRT15, CRABP2, KRT14, HOXB13*
module_2	**GO:0001501**	**skeletal system development**	yes	0.013	6	*HAPLN2, HAPLN3, ACAN, BCAN, IGFBP4, PRELP*
module_2	GO:0007169	transmembrane receptor protein tyrosine kinase signaling pathway	no	0.017	5	*NTRK3, TXK, TEC, NGF, INSRR*
module_2	GO:0007155	cell adhesion	no	0.020	11	*HAPLN2, CDH15, SRPX, HAPLN3, ACAN, BCAN, CNTNAP1, MFGE8, SLAMF1, AOC3, PCDH18*
module_2	**GO:0021570**	**rhombomere 4 development**	yes	0.020	2	*HOXB1, HOXB2*
module_2	GO:0007214	gamma-aminobutyric acid signaling pathway	no	0.021	3	*GABRG1, GABRA2, GABRA4*
module_2	GO:0008306	associative learning	no	0.025	3	*BTG2, NEUROD2, CHRNB2*
module_2	GO:0007165	signal transduction	no	0.029	20	*S100A6, CD244, GIP, STX2, GABRB1, CRABP2, S100A9, S100A11, ARHGAP23, ANXA4, SLAMF1, CD48, SH2D2A, KRT17, PPP1R1B, IL1B, CHRNB2, CNTNAP1, FRS3, IGFBP4*
module_2	GO:1904044	response to aldosterone	no	0.030	2	*CYBA, CYBB*
module_2	GO:0070634	transepithelial ammonium transport	no	0.030	2	*RHCG, RHBG*

module_2	GO:1904845	cellular response to L-glutamine	no	0.030	2	*CYBA*, *CYBB*
module_2	GO:0018146	keratan sulfate biosynthetic process	no	0.033	3	*FMOD*, *ACAN*, *PRELP*
module_2	GO:0006954	inflammatory response	no	0.042	9	*IL17C*, *CYBA*, *CYBB*, *CCR7*, *TSPAN2*, *S100A9*, *IL1B*, *IGFBP4*, *AOC3*
module_2	**GO:0030154**	**cell differentiation**	yes	0.049	10	*ZBTB7B*, *SH2D2A*, *TCP11*, *NR1D1*, *STX2*, *SPDEF*, *TXK*, *NHLH1*, *TEC*, *ETV3*
module_3	GO:0016311	dephosphorylation	no	0.005	3	*NT5C3B*, *PHOSPHO1*, *NT5DC4*
module_3	GO:0071364	cellular response to epidermal growth factor stimulus	no	0.039	2	*ERBB2*, *STAT5B*
module_4	**GO:0030154**	**cell differentiation**	yes	0.005	4	*PPARD*, *ELF2*, *ETV3L*, *ETV4*
module_4	GO:0007264	small GTPase mediated signal transduction	no	0.015	3	*NRAS*, *RAB2B*, *RAB33B*
module_4	GO:0006357	regulation of transcription from RNA polymerase II promoter	no	0.044	3	*PPARD*, *ELF2*, *ETV3L*
module_5	GO:0045893	positive regulation of transcription, DNA-templated	no	0.0000287	16	*CKS1B*, *RAN*, *TAF8*, *PHB*, *SMAD4*, *TFEB*, *NAA15*, *BANP*, *NFYA*, *HMGA1*, *STAT3*, *ARHGEF11*, *ILF2*, *RARA*, *NFE2L1*, *MED1*

module_5	GO:0045471	response to ethanol	no	0.000256	7	*BAK1*, *BGLAP*, *NTRK1*, *RARA*, *RPL10A*, *AACS*, *STAT3*
module_5	GO:0000184	nuclear-transcribed mRNA catabolic process, nonsense-mediated decay	no	0.000502	7	*RPL19*, *RPL23*, *SMG5*, *RPL27*, *RPS10*, *CASC3*, *RPL10A*
module_5	GO:0070102	interleukin-6-mediated signaling pathway	no	0.002	3	*SMAD4*, *IL6R*, *STAT3*
module_5	GO:0046902	regulation of mitochondrial membrane permeability	no	0.003	3	*BAK1*, *CNP*, *STAT3*
module_5	GO:0006412	translation	no	0.006	8	*MRPL24*, *MRPL10*, *RPL19*, *RPL23*, *MRPS11*, *RPL27*, *RPS10*, *RPL10A*
module_5	GO:0070125	mitochondrial translational elongation	no	0.006	5	*MRPL24*, *MRPL10*, *MRPS11*, *MRPL45*, *MRPL46*
module_5	GO:0070126	mitochondrial translational termination	no	0.006	5	*MRPL24*, *MRPL10*, *MRPS11*, *MRPL45*, *MRPL46*
module_5	GO:0006413	translational initiation	no	0.006	6	*RPL19*, *RPL23*, *RPL27*, *RPS10*, *EIF1*, *RPL10A*
module_5	GO:0006614	SRP-dependent cotranslational protein targeting to membrane	no	0.008	5	*RPL19*, *RPL23*, *RPL27*, *RPS10*, *RPL10A*
module_5	GO:0006606	protein import into nucleus	no	0.013	4	*RAN*, *KPNB1*, *STAT3*, *ADAR*
module_5	GO:0019083	viral transcription	no	0.015	5	*RPL19*, *RPL23*, *RPL27*, *RPS10*, *RPL10A*

module_5	GO:0045737	positive regulation of cyclin-dependent protein serine/threonine kinase activity	no	0.017	3	*CKS1B, CDC6, CCND3*
module_5	GO:0006397	mRNA processing	no	0.018	6	*APOBEC2, BCAS2, CDK12, CASC3, SF3B4, ADAR*
module_5	GO:0008285	negative regulation of cell proliferation	no	0.019	9	*CDC6, BAK1, BECN1, NTRK1, PHB, SMAD4, RARA, HMGA1, STAT3*
module_5	GO:0019287	isopentenyl diphosphate biosynthetic process, mevalonate pathway	no	0.025	2	*MVD, PMVK*
module_5	GO:0006914	autophagy	no	0.026	5	*BECN1, SNF8, TFEB, UBQLN4, VPS25*
module_5	GO:0016236	macroautophagy	no	0.026	4	*TOMM6, BECN1, LAMTOR2, ULK1*
module_5	**GO:0035264**	**multicellular organism growth**	yes	0.030	4	*KAT2A, SP2, ANKRD11, RARA*
module_5	GO:0043328	protein targeting to vacuole involved in ubiquitin-dependent protein catabolic process via the multivesicular body sorting pathway	no	0.033	2	*SNF8, VPS25*
module_5	GO:0006364	rRNA processing	no	0.035	6	*RPL19, RPL23, BYSL, RPL27, RPS10, RPL10A*
module_5	GO:0000398	mRNA splicing, via spliceosome	no	0.039	6	*BCAS2, DHX8, FIP1L1, CASC3, SNRPC, SF3B4*
module_5	GO:0006695	cholesterol biosynthetic process	no	0.040	3	*MVD, ACLY, PMVK*

module_5	GO:0016032	viral process	no	0.041	7	*KAT2A, RAN, PSMB3, CALCOCO2, SNAPIN, SHC1, STAT3*
module_5	GO:0042493	response to drug	no	0.043	7	*BAK1, BGLAP, BECN1, NTRK1, DAD1, AACS, STAT3*
module_5	GO:0010508	positive regulation of autophagy	no	0.044	3	*BECN1, ULK1, TFEB*
module_5	**GO:0060348**	**bone development**	yes	0.048	3	*BGLAP, ANKRD11, AKAP13*
module_5	GO:0006886	intracellular protein transport	no	0.049	6	*COPA, RAN, VPS45, SNAPIN, KPNB1, SNX11*
module_5	GO:0000395	mRNA 5'-splice site recognition	no	0.049	2	*SFSWAP, SNRPC*
